# Construction of a detachable artificial trachea model for three age groups for use in an endotracheal suctioning training environment simulator

**DOI:** 10.1371/journal.pone.0249010

**Published:** 2021-03-29

**Authors:** Takaaki Yoshimura, Noriyo Colley, Shunsuke Komizunai, Shinji Ninomiya, Satoshi Kanai, Atsushi Konno, Koichi Yasuda, Hiroshi Taguchi, Takayuki Hashimoto, Shinichi Shimizu

**Affiliations:** 1 Department of Health Sciences and Technology, Faculty of Health Sciences, Hokkaido University, Sapporo, Japan; 2 Department of Medical Physics, Hokkaido University Hospital, Sapporo, Japan; 3 Department of Comprehensive Development Nursing, Faculty of Health Sciences, Hokkaido University, Sapporo, Japan; 4 Division of System Science and Informatics, Graduate School of Information Science and Technology, Hokkaido University, Sapporo, Japan; 5 Department of Medical Science and Technology, Faculty of Health Sciences, Hiroshima International University, Hiroshima, Japan; 6 Department of Radiation Oncology, Faculty of Medicine, Hokkaido University, Sapporo, Japan; 7 Department of Radiation Medical Science and Engineering, Faculty of Medicine, Hokkaido University, Sapporo, Japan; 8 Global Center for Biomedical Science and Engineering, Faculty of Medicine, Hokkaido University, Sapporo, Japan; Juntendo University Urayasu Hospital, JAPAN

## Abstract

Tracheal suctioning is an important procedure to maintain airway patency by removing secretions. Today, suctioning operators include not only medical staff, but also family caregivers. The use of a simulation system has been noted to be the most effective way to learn the tracheal suctioning technique for operators. While the size of the trachea varies across different age groups, the artificial trachea model in the simulation system has only one fixed model. Thus, this study aimed to construct multiple removable trachea models according to different age groups. We enrolled 20 patients who had previously received proton beam therapy in our institution and acquired the treatment planning computed tomography (CT) image data. To construct the artificial trachea model for three age groups (children, adolescents and young adults, and adults), we analyzed the three-dimensional coordinates of the entire trachea, tracheal carina, and the end of the main bronchus. We also analyzed the diameter of the trachea and main bronchus. Finally, we evaluated the accuracy of the model by analyzing the difference between the constructed model and actual measurements. The trachea model was 8 cm long for children and 12 cm for adolescents and young adults, and for adults. The angle between the trachea and bed was about 20 degrees, regardless of age. The mean model accuracy was less than 0.4 cm. We constructed detachable artificial trachea models for three age groups for implementation in the endotracheal suctioning training environment simulator (ESTE-SIM) based on the treatment planning CT image. Our constructed artificial trachea models will be able to provide a simulation environment for various age groups in the ESTE-SIM.

## Introduction

For patients on ventilators, tracheal suctioning to maintain airway patency by removing secretions could be a life-threatening procedure if performed incorrectly. Today, due to the spread of home healthcare services, the operators of the tracheal suctioning include not only medical staff but also family caregivers [1, 2]. However, tracheal suctioning is a highly invasive procedure which involves risk factors such as damage of airway membrane and hemorrhage of tracheoinnominate artery [3–5]. There is also the risk of respiratory obstruction, atelectasis, or aspiration, which increases if the operator hesitates [6, 7]. Simulation education has been attracting attention as an effective way to teach appropriate tracheal suctioning techniques that involve validated principles and rationale [8, 9]. Recently, a simulator has been developed which can quantitatively detect catheter operation in the respiratory tract model [10, 11].

Minimal suction pressure levels, which also avoid Ventilator-associated Pneumonia (VAP), are guided by their viscoelastic properties, such as sputum volume and viscosity [12]. Berra et al. [13] conducted a prospective randomized animal study using intubated sheep. O’ Neal et al. [14] designed a laboratory trachea model which replicated a human trachea and pointed out the necessity to identify safe suction pressures for optimal release of sputum in human subjects.

Although there are some commercially available simulation education systems available for the tracheal suctioning procedure, several clinical situations, such as anatomical changes in the tracheal structure with growth and aging, and instances of incomplete removal of the tracheal secretion, are not addressed, creating a gap between the simulation and clinical practice. To the best of our knowledge, while there are some simulation systems that can be enhanced using biological reactions in the tracheal suctioning procedure, there are some limitations restricting the development of a tracheal suctioning simulator which accounts for varying characteristics of the trachea which are induced by growth and aging. Thus, this study aimed to construct multiple trachea models that can be utilized, depending on the age group of the target subjects.

## Materials and methods

### Patients

This retrospective study was approved by the ethics committee of Hokkaido University Hospital (019–0191) and published on its homepage. A total of 20 patients, who had previously received proton beam therapy and had been given treatment planning computed tomography (CT) for the head and neck region between March 2014 to March 2020 at our institution, were enrolled in this study. The details of the patient characteristics have been given in Table 1. We categorized patients based on age as: children (under 14 years of age); adolescent and young adults (AYA) (15–39 years of age); and adults (over 40 years of age). These are the categories generally used in the field of cancer treatment. All patients had no history of lung surgery and no tracheal tube.

**Table 1:** Patient characteristics.

	Number		Age		Sex	
			Mean	Range	Female	Male
				Min–max		
Total	20	100%	32.1	4.0–75.0	9	11
Children (0~14)	7	35%	7.1	4.0–11.0	2	5
AYA (15~39)	6	30%	25.5	15.0–39.0	2	4
Adult (40~)	7	35%	62.6	44.0–75.0	5	2

AYA = adolescent and young adult

### Endotracheal suctioning training environment simulator

**Fig 1:**
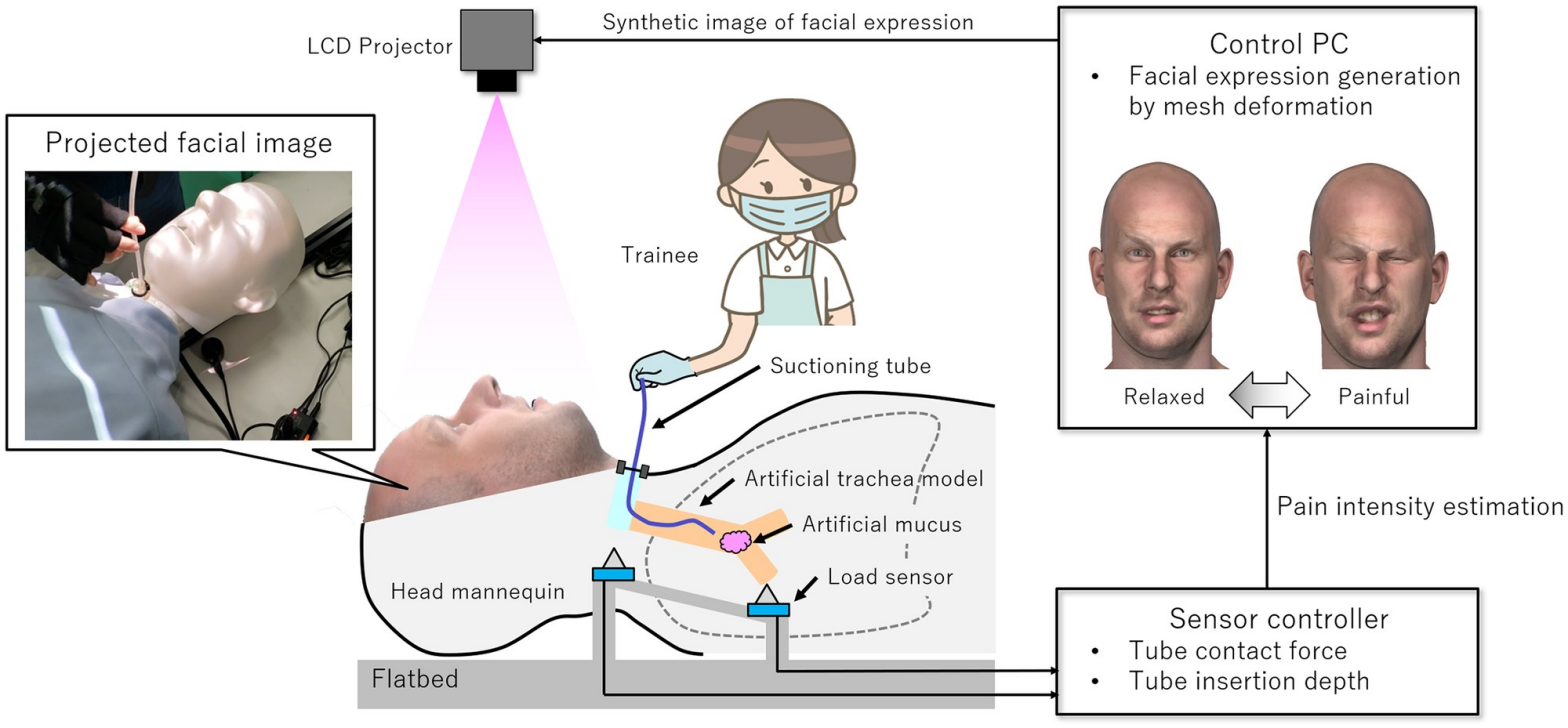
System overview of ESTE-SIM and the artificial trachea model in this study. Projected facial image is a three-dimensional computer graphics (3DCG) model avatar that was purchased and composed from TurboSquid (https://www.turbosquid.com/). ESTE-SIM: endotracheal suctioning training environment simulator, LCD Projector: liquid-crystal display projector, PC: personal computer.

We developed and operated the endotracheal suctioning training environment simulator (ESTE-SIM) which uses interactive vital reactions to change the patient’s condition according to the operator’s procedure (Fig 1) [10]. In this system, it is possible to measure the movement of the suction catheter inserted in the trachea during training, and to study the interactive vital reactions which include not only the monitor-displayed vital signs, but also the real time facial expressions of the patient. This is done through projection mapping based on the actual measurement data of the vital reactions of patients as well as the experimental knowledge of nurses [10]. The artificial trachea model constructed in this study is implemented in ESTE-SIM.

### Modeling

An essential part of the endotracheal suctioning simulator was the artificial trachea model with an anatomically high accuracy. To construct an anatomically accurate artificial trachea model, we used CT images taken for radiation therapy during treatment planning. Unlike the diagnostic CT, the treatment planning CT, which is used for radiation therapy, uses a flatbed as well as endotracheal suction. It is possible to minimize the uncertainty caused by the shape of the bed by using the treatment planning CT images.

**Fig 2:**
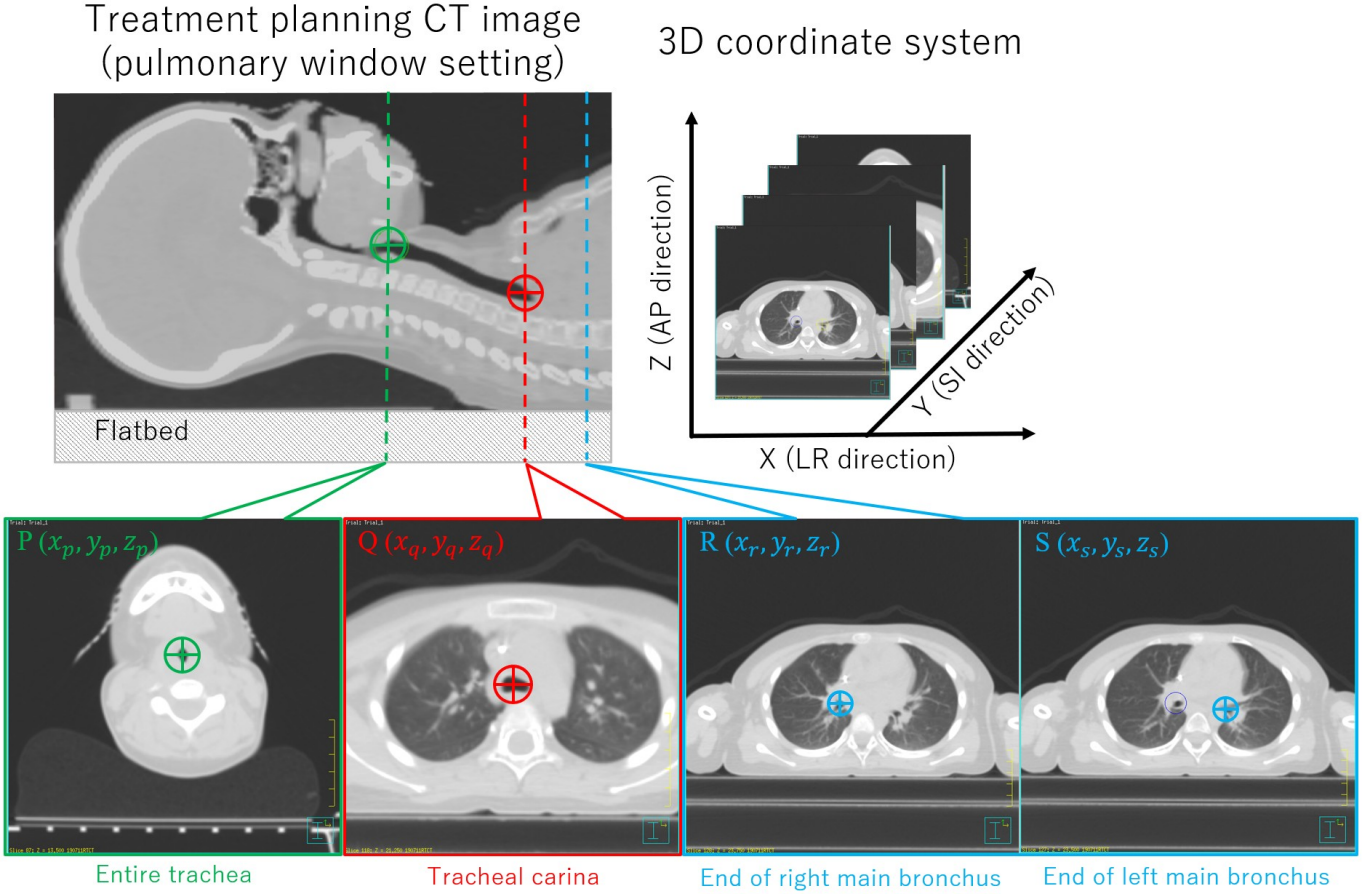
Measurement point in treatment planning CT images.

We defined the measurement points on the treatment planning CT images (Fig 2) which included: the entire trachea (point P (*x*_*p*_, *y*_*p*_, *z*_*p*_)); the tracheal carina, where the trachea joins the main bronchus (point Q (*x*_*q*_, *y*_*q*_, *z*_*q*_)); and, the end of main bronchus (right: point R (*x*_*r*_, *y*_*r*_, *z*_*r*_) and left: point S (*x*_*s*_, *y*_*s*_, *z*_*s*_)). We used the Pinnacle^3^ treatment planning system (TPS; ver.9.1.0, Philips, Inc., Madison, WI) to obtain the three-dimensional coordinates of each evaluation point. To that end, the diameter of the trachea and main bronchus on each slice, as well as the window level of the treatment planning CT image, was set to the pulmonary window setting.

**Fig 3:**
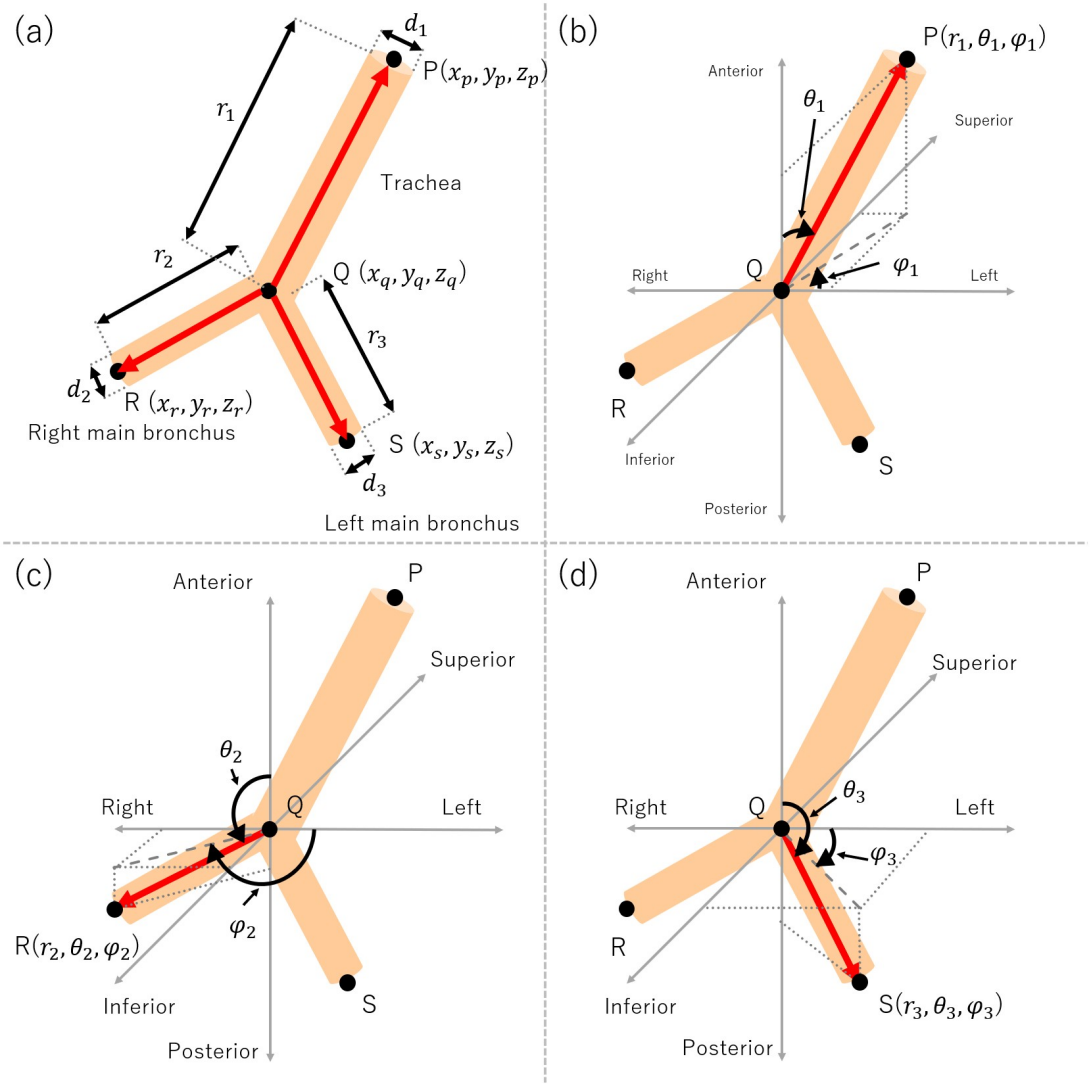
The artificial trachea model parameters in this study. (a) Three-dimensional coordinates of each measurement points and length parameter of trachea and main bronchus, (b) angular parameters for point P, (c) angular parameters for point R, and (d) angular parameters for point S. P: entire trachea, Q: tracheal carina, R: end of the right main bronchus, and S: end of the left main bronchus.

We defined the symbols for modeling the artificial trachea model (Fig 3) which included: *r*_1_, which is the length of trachea; *r*_2_, which is the length of right main bronchus; *r*_3_, which is the length of left main bronchus; *d*_1_, which is the diameter of trachea; *d*_2_, which is the diameter of right main bronchus; and *d*_3_, which is the diameter of left main bronchus. We measured the diameter of trachea (*d*_1_), the right main bronchus (*d*_2_) and the left main bronchus (*d*_3_) on each CT slice. Assuming the artificial trachea model to be cylindrical based on the fractal model [15], we used the average value of diameter in each CT slice.

We normalized the actual three-dimensional coordinates using point Q as a reference point. We defined each normalized point in the three-dimensional coordinates as: point P’ (*x*_*p*′_, *y*_*p*′_, *z*_*p*′_); Q’ (*x*_*q*′_, *y*_*q*′_, *z*_*q*′_); R’ (*x*_*r*′_, *y*_*r*′_, *z*_*r*_); and S’ (*x*_*s*′_, *y*_*s*′_, *z*_*s*′_). Moreover, it was possible to convert the polar coordinates of each point in the three-dimensional coordinates. The coordinates of each point were represented using the angular parameters *θ*_1_, *θ*_2_, *θ*_3_, *φ*_1_, *φ*_2_, and *φ*_3_, where *θ* is the polar angle between the Anterior-Posterior (A-P) direction and each point, and *φ* is the azimuth angle between the Left-Right (L-R) direction and each point. Thus, the modeling parameters for each category (1–13) were calculated using the following formula.

$$\left(\begin{array}{c} x_{p\prime }\\ y_{p\prime }\\ z_{p\prime } \end{array}\right)=\left(\begin{array}{c} x_{p}-x_{q}\\ y_{p}-y_{q}\\ z_{p}-z_{q} \end{array}\right)=\left(\begin{array}{c} r_{1}\;\text{sin}\;\theta_{1}\;\text{cos}\;\varphi_{1}\\ r_{1}\;\text{sin}\;\theta_{1}\;\text{sin}\;\varphi_{1}\\ r_{1}\;\text{cos}\;\theta_{1} \end{array}\right)$$ (1)

$$\left(\begin{array}{c} x_{q\prime }\\ y_{q\prime }\\ z_{q\prime } \end{array}\right)=\left(\begin{array}{c} x_{q}-x_{q}\\ y_{q}-y_{q}\\ z_{q}-z_{q} \end{array}\right)=\left(\begin{array}{c} 0\\ 0\\ 0 \end{array}\right)$$ (2)

$$\left(\begin{array}{c} x_{r\prime }\\ y_{r\prime }\\ z_{r\prime } \end{array}\right)=\left(\begin{array}{c} x_{r}-x_{q}\\ y_{r}-y_{q}\\ z_{r}-z_{q} \end{array}\right)=\left(\begin{array}{c} r_{2}\;\text{sin}\;\theta_{2}\;\text{cos}\;\varphi_{2}\\ r_{2}\;\text{sin}\;\theta_{2}\;\text{sin}\;\varphi_{2}\\ r_{2}\;\text{cos}\;\theta_{2} \end{array}\right)$$ (3)

$$\left(\begin{array}{c} x_{s\prime }\\ y_{s\prime }\\ z_{s\prime } \end{array}\right)=\left(\begin{array}{c} x_{s}-x_{q}\\ y_{s}-y_{q}\\ z_{s}-z_{q} \end{array}\right)=\left(\begin{array}{c} r_{3}\;\text{sin}\;\theta_{3}\;\text{cos}\;\varphi_{3}\\ r_{3}\;\text{sin}\;\theta_{3}\;\text{sin}\;\varphi_{3}\\ r_{3}\;\text{cos}\;\theta_{3} \end{array}\right)$$ (4)

$$r_{1}=|\overset{⃑}{QP}|=\sqrt{{\left(x_{p}-x_{q}\right)}^{2}+{\left(y_{p}-y_{q}\right)}^{2}+{\left(z_{p}-z_{q}\right)}^{2}}$$ (5)

$$r_{2}=|\overset{⃑}{QR}|=\sqrt{{\left(x_{r}-x_{q}\right)}^{2}+{\left(y_{r}-y_{q}\right)}^{2}+{\left(z_{r}-z_{q}\right)}^{2}}$$ (6)

$$r_{3}=|\overset{⃑}{QS}|=\sqrt{{\left(x_{s}-x_{q}\right)}^{2}+{\left(y_{s}-y_{q}\right)}^{2}+{\left(z_{s}-z_{q}\right)}^{2}}$$ (7)

$$\theta_{1}={\text{cos}}^{-1}\;\left(\frac{z_{p}-z_{q}}{r_{1}}\right)$$ (8)

$$\theta_{2}={\text{cos}}^{-1}\;\left(\frac{z_{r}-z_{q}}{r_{2}}\right)$$ (9)

$$\theta_{3}={\text{cos}}^{-1}\;\left(\frac{z_{s}-z_{q}}{r_{2}}\right)$$ (10)

$$\varphi_{1}={\text{cos}}^{-1}\;\left(\frac{x_{p}-x_{q}}{\sqrt{{\left(x_{p}-x_{q}\right)}^{2}+{\left(y_{p}-y_{q}\right)}^{2}}}\right)$$ (11)

$$\varphi_{2}={\text{cos}}^{-1}\;\left(\frac{x_{r}-x_{q}}{\sqrt{{\left(x_{r}-x_{q}\right)}^{2}+{\left(y_{r}-y_{q}\right)}^{2}}}\right)$$ (12)

$$\varphi_{3}={\text{cos}}^{-1}\;\left(\frac{x_{s}-x_{q}}{\sqrt{{\left(x_{s}-x_{q}\right)}^{2}+{\left(y_{s}-y_{q}\right)}^{2}}}\right)$$ (13)

### Model accuracy

We defined each evaluation point of the artificial trachea model constructed in this study as three-dimensional (3D) coordinates which are: point *P*_*m*_ ($x_{p_{m}},y_{p_{m}},\;z_{p_{m}}$); *Q*_*m*_ ($x_{q_{m}},y_{q_{m}},\;z_{q_{m}}$); *R*_*m*_ ($x_{r_{m}},y_{r_{m}},\;z_{r_{m}}$); and *S*_*m*_ ($x_{s_{m}},y_{s_{m}},\;z_{s_{m}}$). To evaluate the model accuracy, we used the difference between the constructed model and actual measurement center coordinates in each slice. Thus, it was seen that the y coordinates were equal ($y_{p_{m}}=y_{p^{'}},\;y_{q_{m}}=y_{q^{'}},\;y_{r_{m}}=y_{r'}$, and $y_{s_{m}}=y_{s'}$). The difference was calculated using following Eqs (14–17).

$$Difference\;\left(point\;P\right)=\left(\begin{array}{c} x_{p_{m}}-x_{p^{\prime }}\\ y_{p_{m}}-y_{p^{\prime }}\\ z_{p_{m}}-z_{p^{\prime }} \end{array}\right)=\left(\begin{array}{c} x_{p_{m}}-x_{p^{\prime }}\\ 0\\ z_{p_{m}}-z_{p^{\prime }} \end{array}\right)$$ (14)

$$Difference\;\left(point\;Q\right)=\left(\begin{array}{c} x_{q_{m}}-x_{q^{\prime }}\\ y_{q_{m}}-y_{q^{\prime }}\\ z_{q_{m}}-z_{q^{\prime }} \end{array}\right)=\left(\begin{array}{c} 0\\ 0\\ 0 \end{array}\right)$$ (15)

$$Difference\;(point\;R)=\left(\begin{array}{c} x_{r_{m}}-x_{r\prime }\\ y_{r_{m}}-y_{r\prime }\\ z_{r_{m}}-z_{r\prime } \end{array}\right)=\left(\begin{array}{c} x_{r_{m}}-x_{r\prime }\\ 0\\ z_{r_{m}}-z_{r\prime } \end{array}\right)$$ (16)

$$Difference\;(point\;S)=\left(\begin{array}{c} x_{s_{m}}-x_{s\prime }\\ y_{s_{m}}-y_{s\prime }\\ z_{s_{m}}-z_{s\prime } \end{array}\right)=\left(\begin{array}{c} x_{s_{m}}-x_{s\prime }\\ 0\\ z_{s_{m}}-z_{s\prime } \end{array}\right)$$ (17)

We used Mann-Whitney’s U test to compare the distribution of the modeling parameters. The Bland-Altman plot was generated to evaluate agreement of the center coordinates in each slice between the constructed artificial trachea model and actual measurement. In the Bland-Altman plot, the horizontal axis shows the mean of the results between the constructed artificial trachea model and actual measurement, whereas the vertical axis represents the difference between the constructed artificial model and actual measurement. The significance level was set at 0.05. All statistical analysis was performed with JMP Pro 14.0.0 (SAS Institute Inc., Cary, NC).

## Results

**Fig 4:**
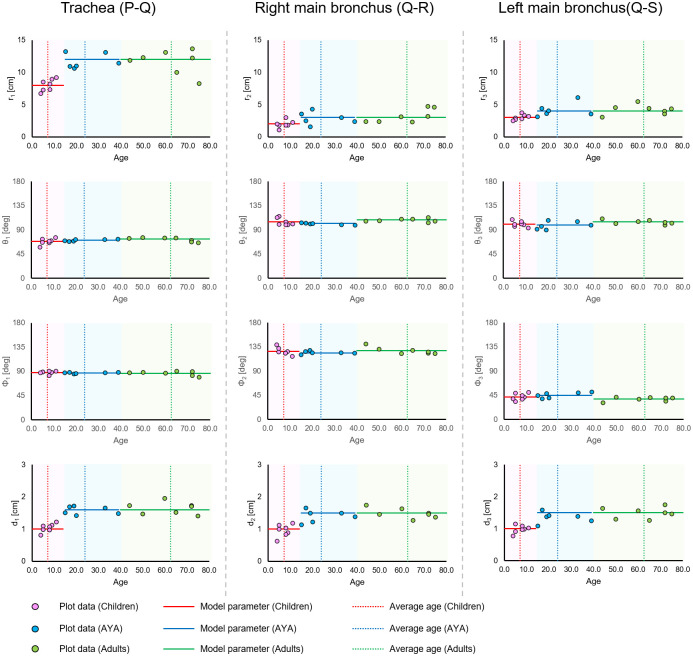
Scatter plot of measurement results as age function. Horizontal axis is patient age; trachea length (*r*_1_), right main bronchus length (*r*_2_), left main bronchus length (*r*_3_), diameter of trachea (*d*_1_), diameter of right main bronchus (*d*_2_), diameter of left main bronchus (*d*_3_), angular parameters in polar coordinates (*θ*_1_, *θ*_2_, *θ*_3_, *φ*_1_, *φ*_2_, *and φ*_3_). AYA: adolescent and young adults.

In this study, we categorized the patients into three age groups (children, AYA, and adults) based on the age categories used in the field of cancer treatment. The average age range and age range for each category can be seen in Table 1. We plotted the artificial model parameters in each patient with the horizontal axis as age, as shown in Fig 4.

As listed in Table 2, we summarized the modeling parameters for each category calculated from the actual coordinates of each evaluation point.

**Table 2:** Summary of results.

		Children	AYA	Adults
		Mean ± S.D.	Mean ± S.D.	Mean ± S.D.
*r*_1_	[cm]	8.08 ± 0.95	11.75 ± 1.14	11.69 ± 1.88
*r*_2_	[cm]	2.02 ± 0.57	2.93 ± 0.95	3.30 ± 1.03
*r*_3_	[cm]	3.10 ± 0.43	4.19 ± 1.05	4.25 ± 0.78
*θ*_1_	[deg]	69.12 ± 5.69	71.46 ± 1.82	72.75 ± 3.90
*θ*_2_	[deg]	105.35 ± 6.59	101.97 ± 1.82	108.82 ± 3.10
*θ*_3_	[deg]	101.50 ± 5.08	98.86 ± 7.21	105.20 ± 4.02
*φ*_1_	[deg]	88.03 ± 2.89	87.10 ± 1.27	86.10 ± 4.10
*φ*_2_	[deg]	126.63 ± 6.95	124.30 ± 2.62	127.65 ± 6.36
*φ*_3_	[deg]	42.31± 6.04	45.36 ± 5.22	38.00 ± 3.90
*d*_1_	[cm]	1.04 ± 0.13	1.58 ± 0.12	1.64 ± 0.19
*d*_2_	[cm]	0.95 ± 0.19	1.40 ± 0.19	1.49 ± 0.16
*d*_3_	[cm]	0.99 ± 0.12	1.35 ± 0.17	1.49 ± 0.17

AYA = adolescent and young adult

We also evaluated the distribution of modeling parameters between age groups. In the length and diameter parameters, there were significant difference between children and the two mature groups. On the other hand, there were no significant differences between AYA and adults in the length and diameter parameters. Moreover, in the angular parameters, there were no significant differences between three age groups except *θ*_2_ between AYA and adults.

**Fig 5:**
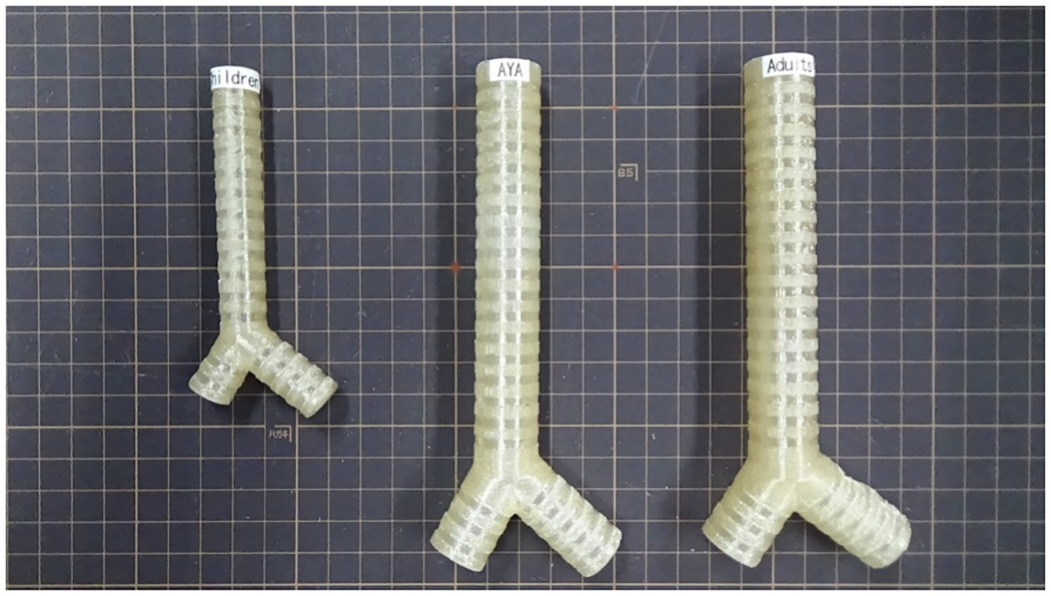
Artificial trachea model for each of the three age groups. Three artificial trachea models created with a 3D printer placed on a 10 mm grid. Left: trachea model for children. Middle: trachea model for adolescents and young adults. Right: trachea model for adults.

We constructed the artificial trachea model using the following parameters for each category based on these results. The parameters of artificial trachea model for children were: *r*_1_ = 8 [cm], *r*_2_ = 2 [cm], *r*_3_ = 3 [cm], *θ*_1_ = 69 [deg], *θ*_2_ = 105 [deg], *θ*_3_ = 102 [deg], *φ*_1_ = 88 [deg], *φ*_2_ = 127 [deg], *φ*_3_ = 42 [deg], *d*_1_ = 1.0 [cm], *d*_2_ = 1.0 [cm], and *d*_3_ = 1.0 [cm]. The parameters of artificial trachea model for AYA were: *r*_1_ = 12 [cm], *r*_2_ = 3 [cm], *r*_3_ = 4 [cm], *θ*_1_ = 71 [deg], *θ*_2_ = 102 [deg], *θ*_3_ = 92 [deg], *φ*_1_ = 87 [deg], *φ*_2_ = 124 [deg], *φ*_3_ = 45 [deg], *d*_1_ = 1.6 [cm], *d*_2_ = 1.5 [cm], and *d*_3_ = 1.5 [cm]. The parameters of artificial trachea model for adults were: *r*_1_ = 12 [cm], *r*_2_ = 3 [cm], *r*_3_ = 4 [cm], *θ*_1_ = 73 [deg], *θ*_2_ = 109 [deg], *θ*_3_ = 128 [deg], *φ*_1_ = 86 [deg], *φ*_2_ = 128 [deg], *φ*_3_ = 38 [deg], *d*_1_ = 1.6 [cm], *d*_2_ = 1.5 [cm], and *d*_3_ = 1.5 [cm]. We created the 3D computer graphic (3DCG) trachea model using a 3D CAD (Computer-Aided Design) software SolidWorks (Dassault Systèmes SolidWorks Corp., Paris, FR) based on these parameters, which was used in our examination. We used a fused deposition modeling (FDM) type 3D printer UP BOX (Tiertime Technology Co. Ltd., Beijing, CN) using acrylonitrile–butadiene–styrene (ABS) resin filament to print three types of artificial trachea models according to the 3D CAD data. The printed artificial trachea models are shown in Fig 5.

**Fig 6:**
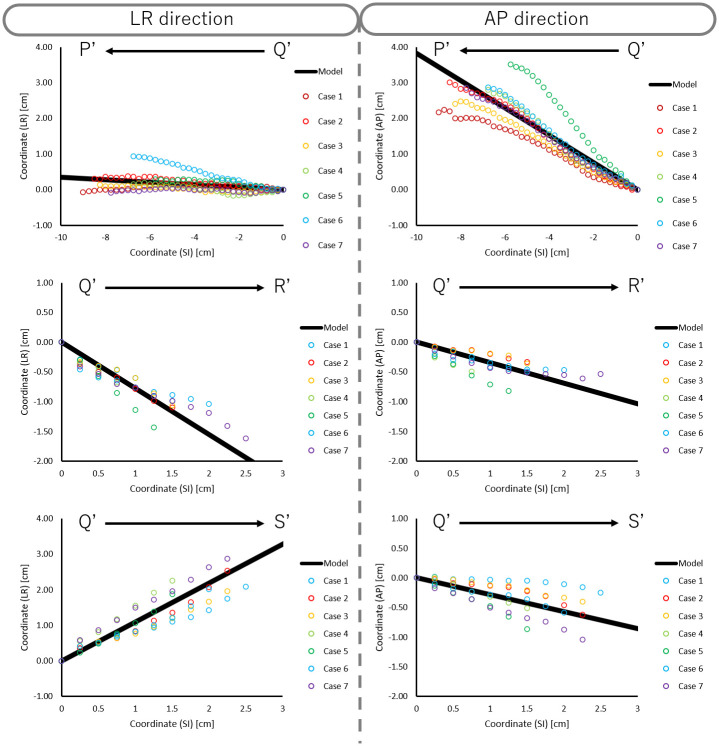
Scatter plot of actual coordinates of the LR and AP directions in each slice and model function for children.

**Fig 7:**
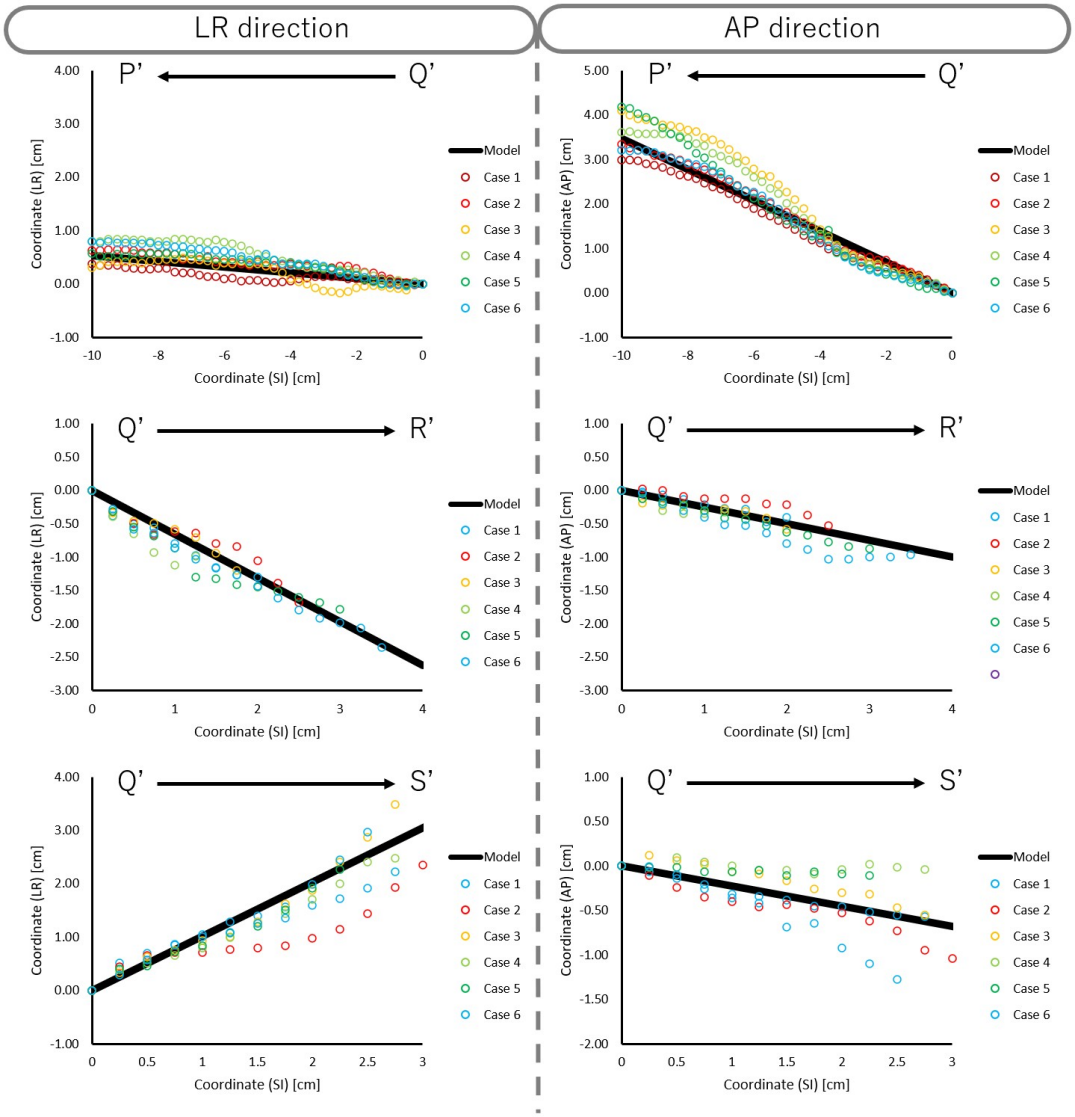
Scatter plot of actual coordinates of the LR and AP directions in each slice and model function for AYA.

**Fig 8:**
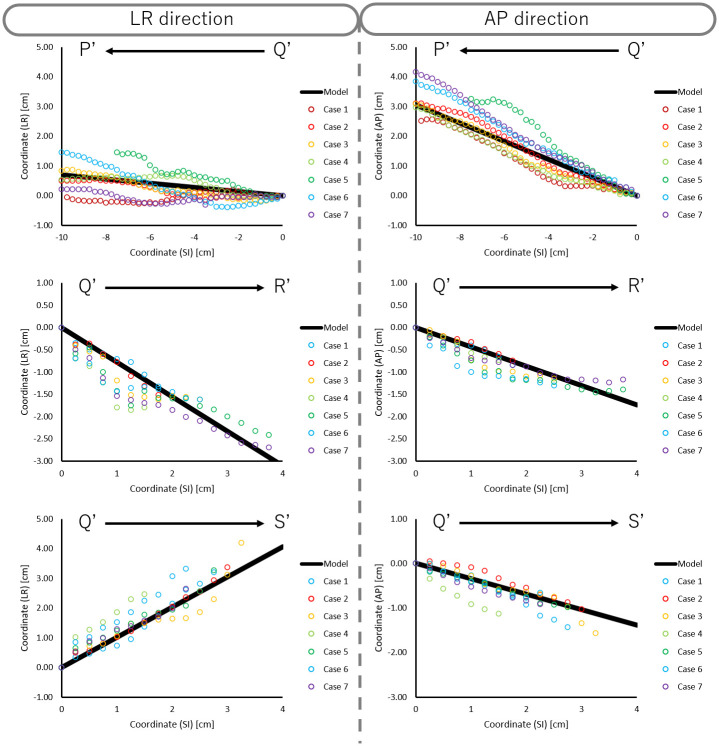
Scatter plot of actual coordinates of the LR and AP directions in each slice and model function for adults.

We plotted the actual coordinates of A-P and L-R direction in each slice, as well as the model function, for children (Fig 6), AYA (Fig 7), and adults (Fig 8). The model accuracy was evaluated using the difference between the center coordinates in constructed artificial trachea model and the actual measurement. The mean and SD of the absolute difference of trachea, right main bronchus, and left main bronchus in A-P and L-R directions are shown in Table 3.

**Table 3:** Summary of modeling accuracy.

	Direction		Children	AYA	Adults
			Mean ± S.D.	Mean ± S.D.	Mean ± S.D.
QP	LR	[cm]	0.14 ± 0.14	0.15 ± 0.11	0.30 ± 0.24
	AP	[cm]	0.29 ± 0.31	0.27 ± 0.23	0.37 ± 0.32
QR	LR	[cm]	0.14 ± 0.13	0.15 ± 0.11	0.28 ± 0.24
	AP	[cm]	0.13 ± 0.12	0.12 ± 0.09	0.16 ± 0.15
QS	LR	[cm]	0.26 ± 0.21	0.36 ± 0.63	0.29 ± 0.29
	AP	[cm]	0.15 ± 0.13	0.24 ± 0.30	0.14 ± 0.15

AYA = adolescent and young adult

**Fig 9:**
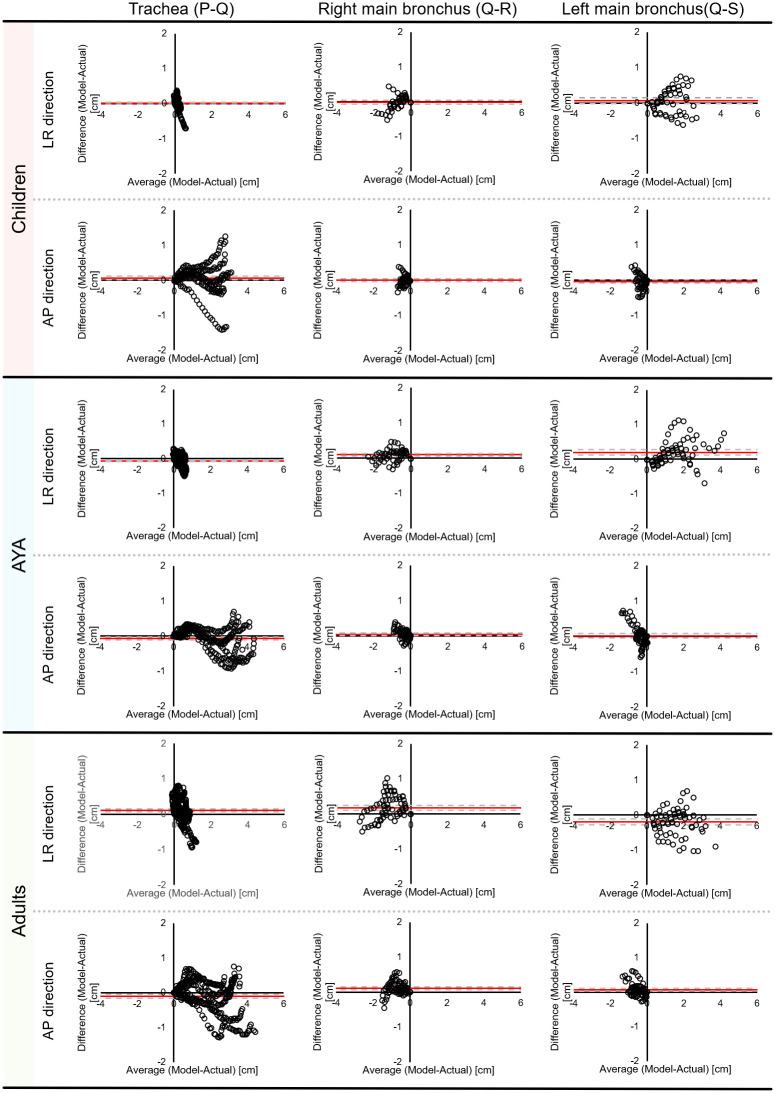
Bland-Altman plot showing the difference between the average of our constructed artificial trachea model and actual measurement. Red solid line denotes the mean of difference and large dashed line denotes 95% limits of agreement.

In the Bland-Altman analysis, mean difference of trachea, right main bronchus, and left main bronchus in A-P direction were 0.01 cm, 0.00 cm, and 0.06 cm for children, -0.07 cm, 0.04 cm, and 0.02 cm for AYA, and -0.10 cm, 0.11 cm, and 0.07 cm for adults. Similarly, mean difference of trachea, right main bronchus, and left main bronchus in L-R direction were 0.01 cm, -0.01 cm, and 0.06 cm for children, -0.07 cm, 0.09 cm, and 0.19 cm for AYA, and 0.11 cm, 0.17 cm, and 0.20 cm for adults. As shown in Fig 9, the scatter of the difference increased progressively as distance from point Q increased.

## Discussion

The main purpose of this study was to represent the artificial trachea model for the educational suctioning simulator. We generalized the artificial trachea model for three different age groups by using the treatment planning CT images taken for proton therapy. The length of trachea model was found to be 8 cm for children and 12 cm for AYA and adults; the length of right main bronchus model was 2 cm for children and 3 cm for AYA and adults; and the length of left main bronchus model was 3 cm for children and 4 cm for AYA and adults. The range of angle between the trachea and bed in the model was 69 to 73 degrees. This suggested that the length of trachea and main bronchus increases from children to AYA, but there is no change in length among AYA and adults. Further, the diameters of the trachea and main bronchus in the model for children were both 1 cm, while they were seen to be 1.6 cm and 1.5 cm in the models for AYA and adults, respectively.

The angle between the trachea and bed is especially important in the simulation of suctioning because this affects the sensation for the operator of inserting the suctioning tube into the patients. To our knowledge, there have been studies that reported the length and diameter of the trachea, but none that study the angles between the bed and trachea [16, 17]. There was no considerable difference in the angle between the tracheal model and bed in each categorized artificial trachea model. The results of this study can be applied to design and construct an artificial trachea model for the endotracheal suctioning simulator.

As shown in Fig 9, the scatter plot of the difference increased progressively as it was farther away from point Q. This result suggested that our constructed artificial trachea model has proportional bias between the constructed artificial trachea model and actual measurement. Also, this indicated that our constructed artificial trachea model has a large error according to the increasing distance between point Q and other points. In order to reduce this error, it is necessary to accumulate more treatment planning CT data, especially in patients under 14 years old.

In our results, as shown in Fig 4, there were no considerable difference in the length and diameter of the trachea and main bronchus between AYA and adults. Although there was no considerable difference in the angular parameters between the children and those over 14 years old, the length and diameter parameters increased lineally by growth in children. Griscom et al., demonstrated the proportion of growth of the trachea by age for people below the age of 20 years using the measurements recorded in CT images [16, 17]. They showed that, among children, the length of trachea increased linearly from 5.4±0.7 cm (0–2 years) to 10.8±1.5 cm (12–14 years), regardless of gender. They also showed that mean diameter of trachea, among children, ranged from 0.53±0.10 cm (0–2 years) to 1.30±0.18 cm (12–14 years) in the A-P direction, and 0.64±0.12 cm (0–2 years) to 1.33±0.16 cm (12–14 years) in the L-R direction. Moreover, Safshekan et al., demonstrated age-related tracheal stiffening [18], which may affect the diameter of trachea. While there is a need to consider growth and stiffening, our constructed artificial trachea model for children does not deviate from previous results. This suggested that it is possible to create 3D models for various age by changing the length and diameter parameters during the 3D printing process.

Previously, there were some commercially available trachea suctioning simulation systems which included biological reaction for adults. Further, these conventional simulation systems had only one trachea model. The ESTE-SIM is a very compact simulation system which allows easy attachment and detachment of trachea models [8–11], that facilitates the preparation of multiple trachea models. The main objective of this study was to construct a trachea model for ESTE-SIM. We constructed three types of the artificial trachea models, according to the age group. As shown in Figs 4–6, our results demonstrated that it is possible to create a model with high accuracy. Objectively and quantitatively accurate artificial trachea models can provide educationally useful information.

This study has certain limitations. The patients in this study have undergone radiation therapy through the usage of a treatment planning CT image and do not require endotracheal suction daily. Thus, this may not accurately reflect the anatomical information of people who require daily endotracheal suction. However, it is difficult to acquire the treatment planning CT image of those patients for the purpose of model construction.

Second, the constructed artificial trachea model assumed that the trachea and main bronchus were cylindrical. The airway of an actual patient has a very complicated shape. In this study, we measured the center coordinate of the trachea and main bronchus and the length in the A-P and L-R directions. The airway does not necessarily form a perfect circle in some slices of the CT images. The main bronchus in particular was remarkable because it ran in the plane of the CT image slice.

Third, all treatment planning CT images were acquired in the supine position on a flat bed. Since endotracheal suction occurs in other positions, variations in shape and angle might occur in different position when suctioning is required.

The other limitation in this study was the limited number of children and AYA patients, particularly since those under 14 years old were categorized as one group. Since the trachea of children grows as they age, it is not accurate to use the artificial trachea model between 4- and 14-years old patients as one model. Although more pediatric patients’ CT images are needed and further data accumulation is desired to construct a more detailed pediatric artificial trachea model, a limited number of pediatric cancer patients were accessible from one facility. The number of pediatric cancer patients whose treatment planning included CT images of the trachea and main bronchus is limited.

## Conclusion

Basic endotracheal suctioning techniques are acquired by repetitive exercises using a simulation system. For realistic suctioning environment simulations for children and adults, it is important to construct an artificial trachea model according to growth and aging. In this study, we constructed an artificial trachea model for three different age categories, children, AYA, and adults, based on treatment planning CT image. Although further research and data accumulation is needed, our results suggested that the use of treatment planning CT images is one methodology that can construct a detachable artificial trachea model based on age for use in various endotracheal suctioning situations.

## Supporting information

S1 File (DOCX)

S2 File (DOCX)
