# Supplementary material for: Construction of a detachable artificial trachea model for three age groups for use in an endotracheal suctioning training environment simulator
Source: PLoS One. 2021 Mar 29;16(3):e0249010. doi: 10.1371/journal.pone.0249010 (PMC8007018; doi:10.1371/journal.pone.0249010)
Supplement: S1 File — (DOCX) [file pone.0249010.s001.docx]

Table 1, Table 2, and Fig 4.

|  | Patient characteristics | | | Modeling parameters | | | | | | | | | | | |
| --- | --- | --- | --- | --- | --- | --- | --- | --- | --- | --- | --- | --- | --- | --- | --- |
| Case No. | Age | Sex | Age group | $r_{1}$ | $\theta_{1}$ | $\varphi_{1}$ | $r_{2}$ | $\theta_{2}$ | $\varphi_{2}$ | $r_{3}$ | $\theta_{3}$ | $\varphi_{3}$ | $d_{1}$ | $d_{2}$ | $d_{3}$ |
| 1 | 11 | F | Children | 9.26 | 76.44 | 90.51 | 2.30 | 101.78 | 117.47 | 3.26 | 94.40 | 50.24 | 1.22 | 1.18 | 1.02 |
| 2 | 9 | M | Children | 9.02 | 70.51 | 87.91 | 1.89 | 100.06 | 126.25 | 3.44 | 100.38 | 41.65 | 1.12 | 0.89 | 0.99 |
| 3 | 5 | M | Children | 8.59 | 73.78 | 89.10 | 1.87 | 101.09 | 125.25 | 3.01 | 97.63 | 48.94 | 0.98 | 0.99 | 0.91 |
| 4 | 5 | F | Children | 7.31 | 67.48 | 88.73 | 1.12 | 115.83 | 132.20 | 2.75 | 100.68 | 33.69 | 1.10 | 1.11 | 1.15 |
| 5 | 4 | M | Children | 6.74 | 58.63 | 87.11 | 2.07 | 113.35 | 138.84 | 2.55 | 109.74 | 38.73 | 0.81 | 0.62 | 0.77 |
| 6 | 8 | M | Children | 7.39 | 67.16 | 82.16 | 1.86 | 105.25 | 123.42 | 2.90 | 101.75 | 44.86 | 1.08 | 1.04 | 0.97 |
| 7 | 8 | M | Children | 8.26 | 69.81 | 90.67 | 3.03 | 100.09 | 122.94 | 3.79 | 105.92 | 38.10 | 0.97 | 0.84 | 1.08 |
| 8 | 39 | F | AYA | 11.48 | 73.50 | 88.13 | 2.42 | 99.75 | 123.02 | 3.58 | 99.16 | 51.09 | 1.48 | 1.39 | 1.25 |
| 9 | 33 | M | AYA | 13.11 | 72.56 | 87.57 | 3.06 | 99.98 | 123.90 | 6.14 | 106.08 | 49.75 | 1.65 | 1.49 | 1.39 |
| 10 | 17 | M | AYA | 10.99 | 68.93 | 88.49 | 2.54 | 103.43 | 125.94 | 4.47 | 97.07 | 38.32 | 1.69 | 1.65 | 1.59 |
| 11 | 19 | M | AYA | 10.67 | 70.16 | 85.31 | 1.63 | 101.69 | 128.38 | 3.70 | 90.62 | 48.07 | 1.71 | 1.49 | 1.37 |
| 12 | 15 | M | AYA | 13.27 | 70.59 | 87.30 | 3.60 | 104.16 | 120.68 | 3.20 | 91.97 | 44.75 | 1.51 | 1.13 | 1.09 |
| 13 | 20 | F | AYA | 11.01 | 72.99 | 85.81 | 4.32 | 102.83 | 123.88 | 4.08 | 108.28 | 40.18 | 1.42 | 1.22 | 1.41 |
| 14 | 65 | F | Adults | 10.07 | 75.51 | 90.35 | 2.38 | 110.65 | 128.26 | 4.46 | 108.72 | 40.67 | 1.52 | 1.27 | 1.27 |
| 15 | 50 | F | Adults | 12.36 | 76.38 | 87.95 | 2.43 | 107.71 | 130.98 | 4.63 | 102.86 | 41.68 | 1.47 | 1.46 | 1.30 |
| 16 | 60 | M | Adults | 13.17 | 75.94 | 86.32 | 3.18 | 110.98 | 122.78 | 5.54 | 106.47 | 37.73 | 1.95 | 1.63 | 1.56 |
| 17 | 44 | M | Adults | 11.92 | 74.97 | 87.61 | 2.45 | 107.30 | 140.19 | 3.10 | 111.36 | 31.27 | 1.73 | 1.74 | 1.63 |
| 18 | 75 | F | Adults | 8.31 | 66.89 | 78.98 | 4.67 | 107.32 | 122.73 | 4.38 | 102.92 | 40.06 | 1.41 | 1.37 | 1.46 |
| 19 | 72 | F | Adults | 12.27 | 71.08 | 82.13 | 3.25 | 113.58 | 122.94 | 4.06 | 99.35 | 34.13 | 1.72 | 1.50 | 1.74 |
| 20 | 72 | F | Adults | 13.71 | 68.47 | 89.37 | 4.76 | 104.23 | 125.65 | 3.59 | 104.70 | 40.44 | 1.71 | 1.46 | 1.49 |
