# Supplementary material for: Construction of a detachable artificial trachea model for three age groups for use in an endotracheal suctioning training environment simulator
Source: PLoS One. 2021 Mar 29;16(3):e0249010. doi: 10.1371/journal.pone.0249010 (PMC8007018; doi:10.1371/journal.pone.0249010)
Supplement: S2 File — (DOCX) [file pone.0249010.s002.docx]

Table 3, Fig 6, Fig 7, Fig 8, and Fig 9.

Center coordinates of trachea in Children (PQ)

| Model | | | Actual | | | | | | | | | | | | | |
| --- | --- | --- | --- | --- | --- | --- | --- | --- | --- | --- | --- | --- | --- | --- | --- | --- |
| Children | | | 1 | | 2 | | 3 | | 4 | | 5 | | 6 | | 7 | |
| L-R | A-P | S-I | L-R | A-P | L-R | A-P | L-R | A-P | L-R | A-P | L-R | A-P | L-R | A-P | L-R | A-P |
| 0.00 | 0.00 | 0.00 | 0.00 | 0.00 | 0.00 | 0.00 | 0.00 | 0.00 | 0.00 | 0.00 | 0.00 | 0.00 | 0.00 | 0.00 | 0.00 | 0.00 |
| 0.01 | 0.10 | -0.25 | 0.00 | 0.04 | 0.00 | 0.00 | -0.02 | 0.09 | -0.05 | 0.06 | -0.03 | 0.11 | 0.02 | 0.12 | -0.04 | 0.08 |
| 0.02 | 0.19 | -0.50 | 0.04 | 0.08 | 0.00 | 0.11 | -0.01 | 0.14 | -0.08 | 0.15 | -0.03 | 0.24 | 0.02 | 0.17 | -0.02 | 0.19 |
| 0.03 | 0.29 | -0.75 | 0.08 | 0.09 | -0.02 | 0.13 | -0.05 | 0.21 | -0.08 | 0.26 | 0.02 | 0.44 | 0.02 | 0.38 | -0.03 | 0.24 |
| 0.03 | 0.38 | -1.00 | 0.10 | 0.15 | 0.00 | 0.22 | -0.07 | 0.21 | -0.09 | 0.32 | 0.09 | 0.54 | 0.07 | 0.32 | -0.03 | 0.30 |
| 0.04 | 0.48 | -1.25 | 0.10 | 0.20 | 0.05 | 0.31 | -0.05 | 0.28 | -0.10 | 0.37 | 0.11 | 0.69 | 0.08 | 0.42 | -0.09 | 0.38 |
| 0.05 | 0.57 | -1.50 | 0.08 | 0.26 | 0.07 | 0.37 | -0.08 | 0.35 | -0.13 | 0.45 | 0.10 | 0.83 | 0.13 | 0.51 | -0.10 | 0.43 |
| 0.06 | 0.67 | -1.75 | 0.08 | 0.29 | 0.09 | 0.46 | -0.06 | 0.42 | -0.16 | 0.52 | 0.11 | 0.91 | 0.20 | 0.57 | -0.09 | 0.49 |
| 0.07 | 0.76 | -2.00 | 0.08 | 0.35 | 0.11 | 0.57 | -0.05 | 0.49 | -0.16 | 0.62 | 0.16 | 1.10 | 0.26 | 0.69 | -0.08 | 0.61 |
| 0.08 | 0.86 | -2.25 | 0.10 | 0.42 | 0.11 | 0.68 | -0.05 | 0.57 | -0.17 | 0.67 | 0.24 | 1.31 | 0.27 | 0.79 | -0.06 | 0.66 |
| 0.09 | 0.96 | -2.50 | 0.10 | 0.53 | 0.14 | 0.77 | -0.07 | 0.65 | -0.15 | 0.74 | 0.25 | 1.51 | 0.31 | 0.88 | -0.02 | 0.76 |
| 0.10 | 1.05 | -2.75 | 0.06 | 0.68 | 0.14 | 0.85 | -0.05 | 0.73 | -0.09 | 0.81 | 0.26 | 1.72 | 0.32 | 0.97 | -0.02 | 0.88 |
| 0.10 | 1.15 | -3.00 | 0.08 | 0.77 | 0.14 | 0.99 | -0.05 | 0.86 | -0.04 | 0.94 | 0.25 | 1.92 | 0.35 | 1.10 | 0.02 | 0.96 |
| 0.11 | 1.24 | -3.25 | 0.15 | 0.86 | 0.18 | 1.10 | -0.05 | 0.95 | 0.02 | 1.06 | 0.24 | 2.12 | 0.40 | 1.27 | -0.01 | 1.09 |
| 0.12 | 1.34 | -3.50 | 0.11 | 0.97 | 0.16 | 1.16 | -0.07 | 1.05 | 0.01 | 1.19 | 0.24 | 2.31 | 0.44 | 1.37 | 0.00 | 1.18 |
| 0.13 | 1.43 | -3.75 | 0.13 | 1.03 | 0.16 | 1.34 | -0.02 | 1.15 | 0.02 | 1.35 | 0.24 | 2.49 | 0.51 | 1.52 | -0.01 | 1.33 |
| 0.14 | 1.53 | -4.00 | 0.13 | 1.12 | 0.20 | 1.42 | 0.01 | 1.23 | 0.06 | 1.57 | 0.28 | 2.65 | 0.56 | 1.66 | -0.02 | 1.42 |
| 0.15 | 1.62 | -4.25 | 0.13 | 1.23 | 0.16 | 1.53 | 0.05 | 1.36 | 0.10 | 1.77 | 0.28 | 2.86 | 0.60 | 1.78 | 0.01 | 1.55 |
| 0.16 | 1.72 | -4.50 | 0.13 | 1.28 | 0.25 | 1.73 | 0.03 | 1.45 | 0.09 | 1.93 | 0.29 | 3.04 | 0.61 | 1.89 | 0.04 | 1.66 |
| 0.17 | 1.82 | -4.75 | 0.13 | 1.36 | 0.27 | 1.86 | 0.03 | 1.52 | 0.11 | 2.05 | 0.28 | 3.19 | 0.69 | 2.05 | 0.05 | 1.83 |
| 0.17 | 1.91 | -5.00 | 0.15 | 1.45 | 0.27 | 1.99 | 0.05 | 1.61 | 0.13 | 2.14 | 0.24 | 3.31 | 0.72 | 2.20 | 0.03 | 1.93 |
| 0.18 | 2.01 | -5.25 | 0.13 | 1.48 | 0.29 | 2.13 | 0.08 | 1.70 | 0.14 | 2.25 | 0.25 | 3.40 | 0.77 | 2.33 | 0.04 | 2.03 |
| 0.19 | 2.10 | -5.50 | 0.17 | 1.59 | 0.31 | 2.24 | 0.07 | 1.82 | 0.12 | 2.36 | 0.28 | 3.44 | 0.80 | 2.43 | 0.02 | 2.15 |
| 0.20 | 2.20 | -5.75 | 0.13 | 1.65 | 0.36 | 2.30 | 0.07 | 1.90 | 0.15 | 2.48 | 0.29 | 3.51 | 0.84 | 2.54 | 0.01 | 2.22 |
| 0.21 | 2.29 | -6.00 | 0.11 | 1.69 | 0.36 | 2.39 | 0.07 | 1.95 | 0.15 | 2.61 |  |  | 0.88 | 2.65 | 0.00 | 2.31 |
| 0.22 | 2.39 | -6.25 | 0.11 | 1.74 | 0.36 | 2.48 | 0.08 | 2.01 | 0.19 | 2.68 |  |  | 0.90 | 2.77 | -0.01 | 2.35 |
| 0.23 | 2.48 | -6.50 | 0.06 | 1.78 | 0.31 | 2.52 | 0.08 | 2.14 | 0.18 | 2.70 |  |  | 0.92 | 2.82 | -0.04 | 2.46 |
| 0.23 | 2.58 | -6.75 | 0.06 | 1.90 | 0.36 | 2.54 | 0.08 | 2.22 | 0.15 | 2.80 |  |  | 0.93 | 2.87 | -0.02 | 2.50 |
| 0.24 | 2.68 | -7.00 | 0.02 | 1.94 | 0.38 | 2.65 | 0.11 | 2.28 |  |  |  |  |  |  | -0.02 | 2.54 |
| 0.25 | 2.77 | -7.25 | 0.00 | 2.00 | 0.33 | 2.70 | 0.09 | 2.29 |  |  |  |  |  |  | -0.03 | 2.58 |
| 0.26 | 2.87 | -7.50 | -0.01 | 2.00 | 0.33 | 2.72 | 0.10 | 2.38 |  |  |  |  |  |  | -0.05 | 2.70 |
| 0.27 | 2.96 | -7.75 | -0.01 | 2.01 | 0.33 | 2.79 | 0.10 | 2.46 |  |  |  |  |  |  | -0.09 | 2.85 |
| 0.28 | 3.06 | -8.00 | -0.01 | 1.98 | 0.36 | 2.83 | 0.09 | 2.47 |  |  |  |  |  |  |  |  |
| 0.29 | 3.15 | -8.25 | -0.01 | 2.00 | 0.29 | 2.94 | 0.13 | 2.40 |  |  |  |  |  |  |  |  |
| 0.30 | 3.25 | -8.50 | -0.03 | 2.20 | 0.31 | 3.01 |  |  |  |  |  |  |  |  |  |  |
| 0.30 | 3.34 | -8.75 | -0.05 | 2.23 |  |  |  |  |  |  |  |  |  |  |  |  |
| 0.31 | 3.44 | -9.00 | -0.08 | 2.17 |  |  |  |  |  |  |  |  |  |  |  |  |

Center coordinates of right main bronchus in Children (QR)

| Model | | | Actual | | | | | | | | | | | | | | | | |
| --- | --- | --- | --- | --- | --- | --- | --- | --- | --- | --- | --- | --- | --- | --- | --- | --- | --- | --- | --- |
| Children | | | 1 | | 2 | | 3 | | 4 | | | 5 | | | 6 | | | 7 | |
| L-R | A-P | S-I | L-R | A-P | L-R | A-P | L-R | A-P | L-R | A-P | L-R | | A-P | L-R | | A-P | L-R | | A-P |
| 0.00 | 0.00 | 0.00 | 0.00 | 0.00 | 0.00 | 0.00 | 0.00 | 0.00 | 0.00 | 0.00 | 0.00 | | 0.00 | 0.00 | | 0.00 | 0.00 | | 0.00 |
| -0.19 | -0.09 | 0.25 | -0.29 | -0.09 | -0.37 | -0.07 | -0.33 | -0.08 | -0.35 | -0.26 | -0.30 | | -0.23 | -0.46 | | -0.20 | -0.41 | | -0.14 |
| -0.39 | -0.17 | 0.50 | -0.42 | -0.16 | -0.50 | -0.13 | -0.40 | -0.14 | -0.48 | -0.38 | -0.57 | | -0.37 | -0.59 | | -0.30 | -0.53 | | -0.24 |
| -0.58 | -0.26 | 0.75 | -0.47 | -0.25 | -0.59 | -0.13 | -0.46 | -0.14 | -0.68 | -0.49 | -0.85 | | -0.56 | -0.64 | | -0.31 | -0.70 | | -0.35 |
| -0.78 | -0.35 | 1.00 | -0.60 | -0.35 | -0.79 | -0.20 | -0.60 | -0.19 |  |  | -1.14 | | -0.71 | -0.76 | | -0.42 | -0.76 | | -0.43 |
| -0.97 | -0.43 | 1.25 | -0.84 | -0.40 | -0.99 | -0.27 | -0.86 | -0.22 |  |  | -1.43 | | -0.82 | -0.90 | | -0.45 | -0.90 | | -0.48 |
| -1.16 | -0.52 | 1.50 | -0.89 | -0.46 | -1.10 | -0.33 | -1.06 | -0.36 |  |  |  | |  | -0.99 | | -0.49 | -0.99 | | -0.51 |
| -1.36 | -0.60 | 1.75 | -0.95 | -0.46 |  |  |  |  |  |  |  | |  |  | |  | -1.09 | | -0.53 |
| -1.55 | -0.69 | 2.00 | -1.04 | -0.47 |  |  |  |  |  |  |  | |  |  | |  | -1.19 | | -0.55 |
| -1.74 | -0.78 | 2.25 |  |  |  |  |  |  |  |  |  | |  |  | |  | -1.41 | | -0.61 |
| -1.94 | -0.86 | 2.50 |  |  |  |  |  |  |  |  |  | |  |  | |  | -1.62 | | -0.53 |

Center coordinates of left main bronchus in Children (QS)

| Model | | | Actual | | | | | | | | | | | | | |
| --- | --- | --- | --- | --- | --- | --- | --- | --- | --- | --- | --- | --- | --- | --- | --- | --- |
| Children | | | 1 | | 2 | | 3 | | 4 | | 5 | | 6 | | 7 | |
| L-R | A-P | S-I | L-R | A-P | L-R | A-P | L-R | A-P | L-R | A-P | L-R | A-P | L-R | A-P | L-R | A-P |
| 0.00 | 0.00 | 0.00 | 0.00 | 0.00 | 0.00 | 0.00 | 0.00 | 0.00 | 0.00 | 0.00 | 0.00 | 0.00 | 0.00 | 0.00 | 0.00 | 0.00 |
| 0.27 | -0.07 | 0.25 | 0.35 | 0.02 | 0.36 | -0.02 | 0.42 | -0.01 | 0.55 | 0.01 | 0.23 | -0.13 | 0.41 | -0.10 | 0.58 | -0.17 |
| 0.55 | -0.14 | 0.50 | 0.52 | -0.02 | 0.51 | -0.09 | 0.54 | -0.03 | 0.81 | -0.11 | 0.49 | -0.25 | 0.52 | -0.18 | 0.86 | -0.26 |
| 0.82 | -0.21 | 0.75 | 0.72 | -0.02 | 0.64 | -0.11 | 0.64 | -0.07 | 1.18 | -0.23 | 0.77 | -0.36 | 0.68 | -0.23 | 1.14 | -0.36 |
| 1.09 | -0.29 | 1.00 | 0.83 | -0.03 | 0.84 | -0.13 | 0.76 | -0.12 | 1.55 | -0.32 | 1.06 | -0.48 | 0.82 | -0.28 | 1.49 | -0.50 |
| 1.37 | -0.36 | 1.25 | 0.94 | -0.05 | 1.13 | -0.16 | 0.96 | -0.13 | 1.92 | -0.42 | 1.39 | -0.65 | 1.00 | -0.29 | 1.72 | -0.59 |
| 1.64 | -0.43 | 1.50 | 1.11 | -0.05 | 1.35 | -0.22 | 1.20 | -0.21 | 2.25 | -0.51 | 1.87 | -0.86 | 1.21 | -0.36 | 1.96 | -0.68 |
| 1.91 | -0.50 | 1.75 | 1.23 | -0.07 | 1.65 | -0.31 | 1.44 | -0.30 |  |  |  |  | 1.54 | -0.48 | 2.28 | -0.74 |
| 2.19 | -0.57 | 2.00 | 1.42 | -0.11 | 2.07 | -0.46 | 1.66 | -0.33 |  |  |  |  | 2.01 | -0.59 | 2.64 | -0.87 |
| 2.46 | -0.64 | 2.25 | 1.75 | -0.16 | 2.53 | -0.62 | 1.96 | -0.40 |  |  |  |  |  |  | 2.87 | -1.04 |
| 2.74 | -0.72 | 2.50 | 2.08 | -0.25 |  |  |  |  |  |  |  |  |  |  |  |  |

Center coordinates of trachea in AYA (PQ)

| Model | | | Actual | | | | | | | | | | | | |
| --- | --- | --- | --- | --- | --- | --- | --- | --- | --- | --- | --- | --- | --- | --- | --- |
| AYA | | | 8 | | 9 | | 10 | | 11 | | 12 | | 13 | | |
| L-R | A-P | S-I | L-R | A-P | L-R | A-P | L-R | A-P | L-R | A-P | L-R | A-P | L-R | A-P |  |
| 0.00 | 0.00 | 0.00 | 0.00 | 0.00 | 0.00 | 0.00 | 0.00 | 0.00 | 0.00 | 0.00 | 0.00 | 0.00 | 0.00 | 0.00 |  |
| 0.01 | 0.09 | -0.25 | 0.00 | 0.10 | -0.02 | 0.11 | 0.00 | 0.06 | 0.04 | 0.08 | 0.00 | 0.02 | -0.01 | 0.05 |  |
| 0.03 | 0.17 | -0.50 | -0.02 | 0.19 | 0.02 | 0.16 | -0.11 | 0.19 | -0.04 | 0.21 | 0.00 | 0.10 | -0.03 | 0.17 |  |
| 0.04 | 0.26 | -0.75 | -0.02 | 0.29 | 0.04 | 0.22 | -0.09 | 0.25 | 0.06 | 0.22 | 0.00 | 0.10 | 0.00 | 0.22 |  |
| 0.05 | 0.35 | -1.00 | 0.02 | 0.40 | 0.09 | 0.36 | -0.07 | 0.37 | 0.06 | 0.32 | 0.00 | 0.14 | -0.01 | 0.28 |  |
| 0.07 | 0.43 | -1.25 | 0.09 | 0.42 | 0.15 | 0.47 | -0.05 | 0.42 | 0.08 | 0.34 | 0.00 | 0.23 | 0.01 | 0.30 |  |
| 0.08 | 0.52 | -1.50 | 0.05 | 0.51 | 0.20 | 0.53 | -0.03 | 0.44 | 0.15 | 0.39 | 0.04 | 0.31 | 0.08 | 0.33 |  |
| 0.09 | 0.61 | -1.75 | 0.07 | 0.55 | 0.22 | 0.62 | -0.05 | 0.46 | 0.21 | 0.47 | 0.08 | 0.39 | 0.12 | 0.38 |  |
| 0.11 | 0.69 | -2.00 | 0.09 | 0.58 | 0.29 | 0.73 | -0.07 | 0.54 | 0.22 | 0.54 | 0.17 | 0.41 | 0.15 | 0.46 |  |
| 0.12 | 0.78 | -2.25 | 0.09 | 0.58 | 0.33 | 0.73 | -0.15 | 0.65 | 0.26 | 0.61 | 0.19 | 0.50 | 0.16 | 0.47 |  |
| 0.13 | 0.87 | -2.50 | 0.13 | 0.64 | 0.33 | 0.80 | -0.17 | 0.69 | 0.26 | 0.63 | 0.23 | 0.54 | 0.21 | 0.52 |  |
| 0.15 | 0.95 | -2.75 | 0.13 | 0.69 | 0.31 | 0.82 | -0.15 | 0.73 | 0.28 | 0.71 | 0.29 | 0.60 | 0.26 | 0.60 |  |
| 0.16 | 1.04 | -3.00 | 0.14 | 0.78 | 0.31 | 0.86 | -0.13 | 0.83 | 0.30 | 0.80 | 0.27 | 0.71 | 0.33 | 0.70 |  |
| 0.17 | 1.13 | -3.25 | 0.13 | 0.82 | 0.31 | 0.95 | -0.07 | 0.88 | 0.32 | 0.91 | 0.31 | 0.83 | 0.38 | 0.80 |  |
| 0.18 | 1.22 | -3.50 | 0.11 | 0.92 | 0.33 | 1.02 | -0.01 | 1.07 | 0.30 | 1.04 | 0.36 | 0.90 | 0.38 | 0.96 |  |
| 0.20 | 1.30 | -3.75 | 0.09 | 0.98 | 0.33 | 1.15 | 0.04 | 1.27 | 0.34 | 1.19 | 0.34 | 1.40 | 0.38 | 1.06 |  |
| 0.21 | 1.39 | -4.00 | 0.05 | 1.12 | 0.38 | 1.22 | 0.10 | 1.42 | 0.34 | 1.32 | 0.40 | 1.29 | 0.35 | 1.18 |  |
| 0.22 | 1.48 | -4.25 | 0.04 | 1.18 | 0.35 | 1.44 | 0.20 | 1.65 | 0.43 | 1.49 | 0.38 | 1.27 | 0.38 | 1.32 |  |
| 0.24 | 1.56 | -4.50 | 0.02 | 1.28 | 0.35 | 1.57 | 0.29 | 1.90 | 0.45 | 1.67 | 0.44 | 1.38 | 0.45 | 1.49 |  |
| 0.25 | 1.65 | -4.75 | 0.04 | 1.43 | 0.35 | 1.68 | 0.33 | 2.09 | 0.52 | 1.86 | 0.40 | 1.54 | 0.56 | 1.61 |  |
| 0.26 | 1.74 | -5.00 | 0.07 | 1.54 | 0.42 | 1.81 | 0.37 | 2.26 | 0.56 | 2.01 | 0.44 | 1.63 | 0.46 | 1.72 |  |
| 0.28 | 1.82 | -5.25 | 0.07 | 1.63 | 0.35 | 1.88 | 0.37 | 2.43 | 0.62 | 2.23 | 0.40 | 1.75 | 0.45 | 1.87 |  |
| 0.29 | 1.91 | -5.50 | 0.05 | 1.72 | 0.38 | 2.01 | 0.41 | 2.61 | 0.71 | 2.34 | 0.46 | 1.88 | 0.51 | 2.04 |  |
| 0.30 | 2.00 | -5.75 | 0.11 | 1.79 | 0.40 | 2.10 | 0.37 | 2.64 | 0.75 | 2.49 | 0.46 | 2.05 | 0.58 | 2.17 |  |
| 0.32 | 2.08 | -6.00 | 0.09 | 1.90 | 0.40 | 2.23 | 0.39 | 2.78 | 0.78 | 2.60 | 0.46 | 2.13 | 0.61 | 2.27 |  |
| 0.33 | 2.17 | -6.25 | 0.13 | 1.99 | 0.42 | 2.30 | 0.35 | 2.97 | 0.82 | 2.73 | 0.46 | 2.28 | 0.62 | 2.28 |  |
| 0.34 | 2.26 | -6.50 | 0.14 | 2.11 | 0.51 | 2.41 | 0.43 | 3.06 | 0.78 | 2.92 | 0.50 | 2.40 | 0.63 | 2.40 |  |
| 0.36 | 2.34 | -6.75 | 0.16 | 2.22 | 0.46 | 2.50 | 0.45 | 3.22 | 0.80 | 2.98 | 0.52 | 2.55 | 0.66 | 2.55 |  |
| 0.37 | 2.43 | -7.00 | 0.20 | 2.33 | 0.46 | 2.58 | 0.46 | 3.33 | 0.82 | 3.07 | 0.55 | 2.72 | 0.66 | 2.66 |  |
| 0.38 | 2.52 | -7.25 | 0.20 | 2.38 | 0.46 | 2.70 | 0.48 | 3.43 | 0.84 | 3.14 | 0.57 | 2.86 | 0.70 | 2.74 |  |
| 0.40 | 2.60 | -7.50 | 0.22 | 2.47 | 0.55 | 2.76 | 0.46 | 3.49 | 0.84 | 3.31 | 0.55 | 3.03 | 0.70 | 2.84 |  |
| 0.41 | 2.69 | -7.75 | 0.29 | 2.56 | 0.58 | 2.83 | 0.46 | 3.62 | 0.80 | 3.35 | 0.57 | 3.13 | 0.72 | 2.89 |  |
| 0.42 | 2.78 | -8.00 | 0.29 | 2.62 | 0.55 | 2.89 | 0.45 | 3.66 | 0.80 | 3.48 | 0.57 | 3.32 | 0.73 | 2.92 |  |
| 0.44 | 2.86 | -8.25 | 0.27 | 2.65 | 0.58 | 2.98 | 0.43 | 3.72 | 0.80 | 3.53 | 0.57 | 3.49 | 0.77 | 2.96 |  |
| 0.45 | 2.95 | -8.50 | 0.27 | 2.74 | 0.58 | 3.03 | 0.39 | 3.75 | 0.82 | 3.57 | 0.55 | 3.57 | 0.75 | 3.02 |  |
| 0.46 | 3.04 | -8.75 | 0.29 | 2.81 | 0.62 | 3.05 | 0.41 | 3.77 | 0.82 | 3.57 | 0.57 | 3.70 | 0.77 | 3.09 |  |
| 0.48 | 3.13 | -9.00 | 0.31 | 2.87 | 0.64 | 3.09 | 0.41 | 3.85 | 0.84 | 3.57 | 0.52 | 3.85 | 0.77 | 3.16 |  |
| 0.49 | 3.21 | -9.25 | 0.34 | 2.90 | 0.64 | 3.18 | 0.41 | 3.89 | 0.84 | 3.57 | 0.55 | 3.95 | 0.77 | 3.18 |  |
| 0.50 | 3.30 | -9.50 | 0.34 | 2.96 | 0.64 | 3.20 | 0.41 | 3.91 | 0.84 | 3.57 | 0.52 | 4.03 | 0.79 | 3.20 |  |
| 0.51 | 3.39 | -9.75 | 0.34 | 2.98 | 0.62 | 3.25 | 0.35 | 4.00 | 0.80 | 3.63 | 0.52 | 4.14 | 0.77 | 3.18 |  |
| 0.53 | 3.47 | -10.00 | 0.38 | 2.98 | 0.62 | 3.34 | 0.31 | 4.10 | 0.80 | 3.61 | 0.57 | 4.18 | 0.79 | 3.20 |  |
| 0.54 | 3.56 | -10.25 | 0.40 | 2.92 | 0.58 | 3.42 | 0.27 | 3.95 | 0.82 | 3.62 | 0.57 | 4.29 | 0.74 | 3.23 |  |
| 0.55 | 3.65 | -10.50 | 0.38 | 2.94 | 0.62 | 3.51 |  |  |  |  | 0.57 | 4.35 | 0.77 | 3.22 |  |
| 0.57 | 3.73 | -10.75 | 0.36 | 3.32 | 0.62 | 3.56 |  |  |  |  | 0.57 | 4.37 |  |  |  |
| 0.58 | 3.82 | -11.00 | 0.36 | 3.26 | 0.62 | 3.62 |  |  |  |  | 0.59 | 4.39 |  |  |  |
| 0.59 | 3.91 | -11.25 |  |  | 0.64 | 3.67 |  |  |  |  | 0.59 | 4.45 |  |  |  |
| 0.61 | 3.99 | -11.50 |  |  | 0.64 | 3.69 |  |  |  |  | 0.59 | 4.49 |  |  |  |
| 0.62 | 4.08 | -11.75 |  |  | 0.60 | 3.75 |  |  |  |  | 0.57 | 4.60 |  |  |  |
| 0.63 | 4.17 | -12.00 |  |  | 0.62 | 3.84 |  |  |  |  | 0.59 | 4.52 |  |  |  |
| 0.65 | 4.25 | -12.25 |  |  | 0.58 | 3.98 |  |  |  |  | 0.55 | 4.47 |  |  |  |
| 0.66 | 4.34 | -12.50 |  |  | 0.53 | 3.93 |  |  |  |  | 0.59 | 4.41 |  |  |  |

Center coordinates of right main bronchus in AYA (QR)

| Model | | | Actual | | | | | | | | | | | |
| --- | --- | --- | --- | --- | --- | --- | --- | --- | --- | --- | --- | --- | --- | --- |
| AYA | | | 8 | | 9 | | 10 | | 11 | | 12 | | 13 | |
| L-R | A-P | S-I | L-R | A-P | L-R | A-P | L-R | A-P | L-R | A-P | L-R | A-P | L-R | A-P |
| 0.00 | 0.00 | 0.00 | 0.00 | 0.00 | 0.00 | 0.00 | 0.00 | 0.00 | 0.00 | 0.00 | 0.00 | 0.00 | 0.00 | 0.00 |
| -0.16 | -0.06 | 0.25 | -0.38 | -0.03 | -0.33 | 0.02 | -0.34 | -0.19 | -0.39 | -0.13 | -0.32 | -0.13 | -0.28 | -0.05 |
| -0.33 | -0.12 | 0.50 | -0.51 | -0.07 | -0.49 | 0.00 | -0.46 | -0.23 | -0.65 | -0.30 | -0.55 | -0.17 | -0.58 | -0.20 |
| -0.49 | -0.19 | 0.75 | -0.56 | -0.14 | -0.66 | -0.09 | -0.49 | -0.23 | -0.93 | -0.35 | -0.69 | -0.21 | -0.64 | -0.30 |
| -0.66 | -0.25 | 1.00 | -0.80 | -0.21 | -0.62 | -0.13 | -0.59 | -0.30 | -1.12 | -0.35 | -0.86 | -0.30 | -0.86 | -0.41 |
| -0.82 | -0.31 | 1.25 | -0.98 | -0.27 | -0.64 | -0.13 | -0.71 | -0.28 | -0.99 | -0.33 | -1.30 | -0.42 | -1.03 | -0.52 |
| -0.98 | -0.37 | 1.50 | -1.16 | -0.28 | -0.80 | -0.13 | -0.94 | -0.32 |  |  | -1.32 | -0.44 | -1.17 | -0.53 |
| -1.15 | -0.44 | 1.75 | -1.27 | -0.47 | -0.84 | -0.20 | -1.20 | -0.42 |  |  | -1.41 | -0.53 | -1.27 | -0.64 |
| -1.31 | -0.50 | 2.00 | -1.30 | -0.41 | -1.06 | -0.22 | -1.45 | -0.59 |  |  | -1.45 | -0.63 | -1.44 | -0.80 |
| -1.48 | -0.56 | 2.25 |  |  | -1.39 | -0.37 |  |  |  |  | -1.51 | -0.67 | -1.61 | -0.89 |
| -1.64 | -0.62 | 2.50 |  |  | -1.68 | -0.53 |  |  |  |  | -1.60 | -0.78 | -1.80 | -1.03 |
| -1.81 | -0.69 | 2.75 |  |  |  |  |  |  |  |  | -1.68 | -0.84 | -1.92 | -1.03 |
| -1.97 | -0.75 | 3.00 |  |  |  |  |  |  |  |  | -1.78 | -0.88 | -1.98 | -1.00 |
| -2.13 | -0.81 | 3.25 |  |  |  |  |  |  |  |  |  |  | -2.06 | -1.00 |
| -2.30 | -0.87 | 3.50 |  |  |  |  |  |  |  |  |  |  | -2.35 | -0.96 |

Center coordinates of left main bronchus in AYA (QS)

| Model | | | Actual | | | | | | | | | | | | | |
| --- | --- | --- | --- | --- | --- | --- | --- | --- | --- | --- | --- | --- | --- | --- | --- | --- |
| AYA | | | 8 | | 9 | | 10 | | 11 | | 12 | | | 13 | | |
| L-R | A-P | S-I | L-R | A-P | L-R | A-P | L-R | A-P | L-R | A-P | L-R | A-P | L-R | | A-P |  |
| 0.00 | 0.00 | 0.00 | 0.00 | 0.00 | 0.00 | 0.00 | 0.00 | 0.00 | 0.00 | 0.00 | 0.00 | 0.00 | 0.00 | | 0.00 |  |
| 0.25 | -0.06 | 0.25 | 0.52 | -0.07 | 0.44 | -0.11 | 0.33 | 0.12 | 0.39 | -0.02 | 0.40 | -0.02 | 0.29 | | -0.01 |  |
| 0.51 | -0.11 | 0.50 | 0.69 | -0.14 | 0.66 | -0.24 | 0.62 | 0.06 | 0.52 | 0.09 | 0.46 | -0.02 | 0.57 | | -0.10 |  |
| 0.76 | -0.17 | 0.75 | 0.87 | -0.21 | 0.71 | -0.35 | 0.75 | 0.02 | 0.65 | 0.04 | 0.73 | -0.07 | 0.85 | | -0.26 |  |
| 1.02 | -0.23 | 1.00 | 1.05 | -0.32 | 0.71 | -0.40 | 0.93 | -0.07 | 0.80 | 0.00 | 0.84 | -0.07 | 1.00 | | -0.36 |  |
| 1.27 | -0.28 | 1.25 | 1.29 | -0.34 | 0.77 | -0.46 | 1.00 | -0.09 | 0.99 | -0.05 | 1.07 | -0.05 | 1.09 | | -0.42 |  |
| 1.52 | -0.34 | 1.50 | 1.39 | -0.39 | 0.80 | -0.44 | 1.29 | -0.17 | 1.25 | -0.05 | 1.20 | -0.11 | 1.25 | | -0.69 |  |
| 1.78 | -0.40 | 1.75 | 1.57 | -0.45 | 0.84 | -0.48 | 1.62 | -0.26 | 1.44 | -0.09 | 1.51 | -0.07 | 1.36 | | -0.65 |  |
| 2.03 | -0.46 | 2.00 | 1.59 | -0.46 | 0.97 | -0.53 | 1.87 | -0.30 | 1.70 | -0.04 | 1.91 | -0.09 | 1.99 | | -0.92 |  |
| 2.29 | -0.51 | 2.25 | 1.72 | -0.52 | 1.15 | -0.62 | 2.40 | -0.32 | 2.00 | 0.02 | 2.27 | -0.11 | 2.44 | | -1.10 |  |
| 2.54 | -0.57 | 2.50 | 1.91 | -0.55 | 1.44 | -0.73 | 2.86 | -0.47 | 2.41 | -0.02 |  |  | 2.96 | | -1.28 |  |
| 2.79 | -0.63 | 2.75 | 2.22 | -0.57 | 1.93 | -0.95 | 3.48 | -0.55 | 2.47 | -0.04 |  |  |  | |  |  |
| 3.05 | -0.68 | 3.00 |  |  | 2.35 | -1.04 |  |  |  |  |  |  |  | |  |  |
| 3.30 | -0.74 | 3.25 |  |  | 2.86 | -1.19 |  |  |  |  |  |  |  | |  |  |
| 3.56 | -0.80 | 3.50 |  |  | 3.23 | -1.32 |  |  |  |  |  |  |  | |  |  |
| 3.81 | -0.85 | 3.75 |  |  | 3.56 | -1.52 |  |  |  |  |  |  |  | |  |  |
| 4.06 | -0.91 | 4.00 |  |  | 3.68 | -1.65 |  |  |  |  |  |  |  | |  |  |
| 4.32 | -0.97 | 4.25 |  |  | 3.74 | -1.63 |  |  |  |  |  |  |  | |  |  |
| 4.57 | -1.02 | 4.50 |  |  | 3.81 | -1.70 |  |  |  |  |  |  |  | |  |  |

Center coordinates of trachea in Adults (PQ)

| Model | | | Actual | | | | | | | | | | | | | | |
| --- | --- | --- | --- | --- | --- | --- | --- | --- | --- | --- | --- | --- | --- | --- | --- | --- | --- |
| Adults | | | 14 | | 15 | | 16 | | 17 | | 18 | | 19 | | 20 | |  |
| L-R | A-P | S-I | L-R | A-P | L-R | A-P | L-R | A-P | L-R | A-P | L-R | A-P | L-R | A-P | L-R | A-P |  |
| 0.00 | 0.00 | 0.00 | 0.00 | 0.00 | 0.00 | 0.00 | 0.00 | 0.00 | 0.00 | 0.00 | 0.00 | 0.00 | 0.00 | 0.00 | 0.00 | 0.00 |  |
| 0.02 | 0.08 | -0.25 | -0.02 | 0.07 | 0.00 | 0.05 | -0.02 | 0.09 | -0.06 | 0.07 | -0.04 | 0.05 | -0.10 | 0.13 | -0.05 | 0.19 |  |
| 0.03 | 0.15 | -0.50 | 0.00 | 0.12 | 0.00 | 0.08 | -0.09 | 0.11 | -0.10 | 0.09 | -0.01 | 0.06 | -0.12 | 0.28 | -0.06 | 0.28 |  |
| 0.05 | 0.23 | -0.75 | 0.00 | 0.16 | 0.00 | 0.22 | -0.18 | 0.11 | -0.05 | 0.04 | -0.01 | 0.17 | -0.16 | 0.30 | -0.04 | 0.30 |  |
| 0.07 | 0.31 | -1.00 | 0.00 | 0.19 | -0.04 | 0.22 | -0.18 | 0.18 | -0.03 | 0.16 | 0.00 | 0.33 | -0.21 | 0.52 | -0.01 | 0.38 |  |
| 0.09 | 0.38 | -1.25 | -0.02 | 0.24 | 0.00 | 0.29 | -0.15 | 0.24 | -0.03 | 0.22 | 0.05 | 0.52 | -0.27 | 0.55 | 0.04 | 0.42 |  |
| 0.10 | 0.46 | -1.50 | 0.04 | 0.22 | -0.02 | 0.34 | -0.15 | 0.31 | -0.02 | 0.26 | 0.15 | 0.61 | -0.29 | 0.53 | -0.05 | 0.53 |  |
| 0.12 | 0.53 | -1.75 | 0.07 | 0.29 | -0.04 | 0.44 | -0.15 | 0.31 | 0.00 | 0.27 | 0.20 | 0.70 | -0.34 | 0.59 | -0.05 | 0.58 |  |
| 0.14 | 0.61 | -2.00 | 0.14 | 0.33 | -0.07 | 0.46 | -0.13 | 0.39 | 0.00 | 0.35 | 0.27 | 0.84 | -0.35 | 0.66 | -0.04 | 0.78 |  |
| 0.16 | 0.69 | -2.25 | 0.19 | 0.33 | -0.04 | 0.56 | -0.13 | 0.46 | 0.03 | 0.45 | 0.37 | 0.93 | -0.38 | 0.79 | -0.05 | 0.87 |  |
| 0.17 | 0.76 | -2.50 | 0.16 | 0.31 | -0.04 | 0.61 | -0.09 | 0.46 | 0.11 | 0.46 | 0.49 | 1.03 | -0.39 | 0.78 | -0.04 | 1.02 |  |
| 0.19 | 0.84 | -2.75 | 0.14 | 0.33 | -0.04 | 0.66 | -0.02 | 0.52 | 0.16 | 0.52 | 0.48 | 1.17 | -0.38 | 0.90 | -0.02 | 1.07 |  |
| 0.21 | 0.92 | -3.00 | 0.11 | 0.31 | -0.07 | 0.71 | -0.02 | 0.59 | 0.26 | 0.53 | 0.53 | 1.24 | -0.38 | 1.09 | -0.06 | 1.12 |  |
| 0.23 | 0.99 | -3.25 | 0.12 | 0.31 | -0.07 | 0.73 | 0.02 | 0.65 | 0.28 | 0.55 | 0.55 | 1.33 | -0.24 | 1.15 | -0.09 | 1.23 |  |
| 0.24 | 1.07 | -3.50 | 0.12 | 0.42 | -0.14 | 0.78 | 0.04 | 0.74 | 0.37 | 0.60 | 0.61 | 1.45 | -0.21 | 1.30 | -0.31 | 1.27 |  |
| 0.26 | 1.14 | -3.75 | 0.12 | 0.47 | -0.04 | 0.92 | 0.08 | 0.82 | 0.43 | 0.63 | 0.64 | 1.64 | -0.16 | 1.43 | -0.15 | 1.33 |  |
| 0.28 | 1.22 | -4.00 | 0.09 | 0.54 | -0.04 | 1.00 | 0.11 | 0.89 | 0.51 | 0.69 | 0.69 | 1.88 | -0.07 | 1.49 | -0.13 | 1.43 |  |
| 0.30 | 1.30 | -4.25 | 0.09 | 0.63 | 0.03 | 1.09 | 0.15 | 0.98 | 0.59 | 0.83 | 0.76 | 2.05 | 0.03 | 1.59 | -0.15 | 1.52 |  |
| 0.31 | 1.37 | -4.50 | 0.00 | 0.77 | 0.03 | 1.29 | 0.13 | 1.00 | 0.63 | 0.89 | 0.84 | 2.32 | 0.02 | 1.63 | -0.20 | 1.63 |  |
| 0.33 | 1.45 | -4.75 | -0.05 | 0.90 | 0.03 | 1.41 | 0.17 | 1.04 | 0.66 | 0.99 | 0.80 | 2.48 | 0.08 | 1.75 | -0.20 | 1.78 |  |
| 0.35 | 1.53 | -5.00 | -0.12 | 1.01 | 0.05 | 1.50 | 0.15 | 1.13 | 0.66 | 1.05 | 0.73 | 2.55 | 0.12 | 1.74 | -0.23 | 1.87 |  |
| 0.37 | 1.60 | -5.25 | -0.16 | 1.13 | 0.08 | 1.65 | 0.15 | 1.26 | 0.64 | 1.18 | 0.72 | 2.80 | 0.18 | 1.90 | -0.28 | 1.94 |  |
| 0.38 | 1.68 | -5.50 | -0.21 | 1.24 | 0.15 | 1.75 | 0.15 | 1.43 | 0.66 | 1.29 | 0.77 | 2.88 | 0.21 | 2.01 | -0.28 | 2.04 |  |
| 0.40 | 1.75 | -5.75 | -0.26 | 1.36 | 0.22 | 1.87 | 0.21 | 1.58 | 0.67 | 1.35 | 0.87 | 3.03 | 0.29 | 2.11 | -0.27 | 2.21 |  |
| 0.42 | 1.83 | -6.00 | -0.23 | 1.43 | 0.29 | 1.99 | 0.26 | 1.69 | 0.64 | 1.46 | 1.02 | 3.12 | 0.40 | 2.23 | -0.27 | 2.33 |  |
| 0.44 | 1.91 | -6.25 | -0.26 | 1.60 | 0.34 | 2.11 | 0.30 | 1.84 | 0.61 | 1.59 | 1.27 | 3.16 | 0.46 | 2.38 | -0.24 | 2.44 |  |
| 0.45 | 1.98 | -6.50 | -0.28 | 1.69 | 0.37 | 2.20 | 0.36 | 1.97 | 0.59 | 1.68 | 1.34 | 3.23 | 0.57 | 2.50 | -0.23 | 2.54 |  |
| 0.47 | 2.06 | -6.75 | -0.24 | 1.79 | 0.42 | 2.33 | 0.45 | 2.08 | 0.54 | 1.78 | 1.40 | 3.15 | 0.67 | 2.58 | -0.15 | 2.71 |  |
| 0.49 | 2.14 | -7.00 | -0.23 | 1.85 | 0.44 | 2.45 | 0.49 | 2.16 | 0.57 | 1.87 | 1.43 | 3.14 | 0.69 | 2.71 | -0.14 | 2.84 |  |
| 0.50 | 2.21 | -7.25 | -0.23 | 1.95 | 0.44 | 2.57 | 0.56 | 2.27 | 0.56 | 1.97 | 1.39 | 3.16 | 0.71 | 2.82 | -0.09 | 2.95 |  |
| 0.52 | 2.29 | -7.50 | -0.21 | 2.06 | 0.49 | 2.64 | 0.62 | 2.38 | 0.60 | 2.04 | 1.46 | 3.26 | 0.87 | 2.88 | -0.04 | 3.07 |  |
| 0.54 | 2.36 | -7.75 | -0.21 | 2.14 | 0.49 | 2.71 | 0.65 | 2.42 | 0.63 | 2.12 |  |  | 0.96 | 3.10 | 0.05 | 3.22 |  |
| 0.56 | 2.44 | -8.00 | -0.24 | 2.23 | 0.49 | 2.78 | 0.69 | 2.49 | 0.60 | 2.21 |  |  | 0.99 | 3.16 | 0.10 | 3.38 |  |
| 0.57 | 2.52 | -8.25 | -0.17 | 2.28 | 0.51 | 2.81 | 0.69 | 2.51 | 0.60 | 2.30 |  |  | 1.07 | 3.28 | 0.12 | 3.53 |  |
| 0.59 | 2.59 | -8.50 | -0.17 | 2.37 | 0.56 | 2.86 | 0.71 | 2.57 | 0.60 | 2.37 |  |  | 1.12 | 3.40 | 0.13 | 3.63 |  |
| 0.61 | 2.67 | -8.75 | -0.19 | 2.46 | 0.51 | 2.93 | 0.75 | 2.66 | 0.59 | 2.50 |  |  | 1.20 | 3.49 | 0.21 | 3.74 |  |
| 0.63 | 2.75 | -9.00 | -0.17 | 2.51 | 0.51 | 2.91 | 0.75 | 2.63 | 0.57 | 2.61 |  |  | 1.29 | 3.50 | 0.22 | 3.83 |  |
| 0.64 | 2.82 | -9.25 | -0.16 | 2.56 | 0.49 | 2.98 | 0.80 | 2.85 | 0.57 | 2.73 |  |  | 1.34 | 3.62 | 0.22 | 3.94 |  |
| 0.66 | 2.90 | -9.50 | -0.12 | 2.56 | 0.49 | 3.03 | 0.86 | 2.94 | 0.56 | 2.87 |  |  | 1.37 | 3.65 | 0.22 | 4.00 |  |
| 0.68 | 2.97 | -9.75 | -0.06 | 2.52 | 0.49 | 3.10 | 0.86 | 3.03 | 0.53 | 2.91 |  |  | 1.44 | 3.72 | 0.21 | 4.06 |  |
| 0.70 | 3.05 | -10.00 |  |  | 0.49 | 3.10 | 0.84 | 3.09 | 0.53 | 2.96 |  |  | 1.45 | 3.84 | 0.22 | 4.16 |  |
| 0.71 | 3.13 | -10.25 |  |  | 0.51 | 3.07 | 0.86 | 3.11 | 0.50 | 3.01 |  |  | 1.46 | 3.88 | 0.22 | 4.33 |  |
| 0.73 | 3.20 | -10.50 |  |  | 0.56 | 3.03 | 0.86 | 3.07 | 0.53 | 3.04 |  |  | 1.48 | 3.94 | 0.22 | 4.41 |  |
| 0.75 | 3.28 | -10.75 |  |  | 0.56 | 2.98 | 0.82 | 3.05 | 0.50 | 3.06 |  |  | 1.46 | 3.97 | 0.23 | 4.48 |  |
| 0.77 | 3.36 | -11.00 |  |  | 0.51 | 2.98 | 0.82 | 3.05 | 0.43 | 3.11 |  |  | 1.53 | 3.96 | 0.21 | 4.56 |  |
| 0.78 | 3.43 | -11.25 |  |  | 0.51 | 3.10 | 0.80 | 3.09 | 0.48 | 3.06 |  |  | 1.56 | 3.96 | 0.23 | 4.60 |  |
| 0.80 | 3.51 | -11.50 |  |  | 0.46 | 3.12 | 0.80 | 3.09 | 0.48 | 3.09 |  |  | 1.59 | 3.98 | 0.25 | 4.61 |  |
| 0.82 | 3.58 | -11.75 |  |  | 0.44 | 3.03 | 0.78 | 3.14 |  |  |  |  |  |  | 0.23 | 4.68 |  |
| 0.84 | 3.66 | -12.00 |  |  | 0.43 | 2.91 | 0.86 | 3.48 |  |  |  |  |  |  | 0.26 | 4.64 |  |
| 0.85 | 3.74 | -12.25 |  |  |  |  | 0.82 | 3.48 |  |  |  |  |  |  | 0.25 | 4.65 |  |
| 0.87 | 3.81 | -12.50 |  |  |  |  | 0.78 | 3.35 |  |  |  |  |  |  | 0.25 | 4.90 |  |
| 0.89 | 3.89 | -12.75 |  |  |  |  | 0.82 | 3.20 |  |  |  |  |  |  | 0.14 | 5.03 |  |

Center coordinates of right main bronchus in Adults (QR)

| Model | | | Actual | | | | | | | | | | | | | | |
| --- | --- | --- | --- | --- | --- | --- | --- | --- | --- | --- | --- | --- | --- | --- | --- | --- | --- |
| Adults | | | 14 | | 15 | | 16 | | 17 | | 18 | | 19 | | 20 | |  |
| L-R | A-P | S-I | L-R | A-P | L-R | A-P | L-R | A-P | L-R | A-P | L-R | A-P | L-R | A-P | L-R | A-P |  |
| 0.00 | 0.00 | 0.00 | 0.00 | 0.00 | 0.00 | 0.00 | 0.00 | 0.00 | 0.00 | 0.00 | 0.00 | 0.00 | 0.00 | 0.00 | 0.00 | 0.00 |  |
| -0.19 | -0.11 | 0.25 | -0.35 | -0.23 | -0.38 | -0.16 | -0.41 | -0.06 | -0.69 | -0.17 | -0.59 | -0.22 | -0.70 | -0.40 | -0.49 | -0.24 |  |
| -0.39 | -0.22 | 0.50 | -0.49 | -0.33 | -0.36 | -0.19 | -0.54 | -0.19 | -0.86 | -0.23 | -0.45 | -0.40 | -0.81 | -0.47 | -0.68 | -0.34 |  |
| -0.58 | -0.33 | 0.75 | -0.59 | -0.37 | -0.60 | -0.26 | -0.65 | -0.32 | -1.22 | -0.40 | -1.00 | -0.58 | -1.24 | -0.87 | -1.13 | -0.50 |  |
| -0.78 | -0.43 | 1.00 | -0.71 | -0.40 | -0.77 | -0.33 | -1.19 | -0.73 | -1.80 | -0.57 | -1.43 | -0.74 | -1.44 | -1.00 | -1.54 | -0.68 |  |
| -0.97 | -0.54 | 1.25 | -0.77 | -0.51 | -1.09 | -0.48 | -1.52 | -0.90 | -1.85 | -0.66 | -1.75 | -1.01 | -1.36 | -1.09 | -1.63 | -0.74 |  |
| -1.16 | -0.65 | 1.50 | -1.06 | -0.66 | -1.31 | -0.60 | -1.56 | -0.99 | -1.80 | -0.73 | -1.43 | -0.98 | -1.34 | -1.09 | -1.69 | -0.77 |  |
| -1.36 | -0.76 | 1.75 | -1.38 | -0.84 | -1.52 | -0.74 | -1.61 | -0.99 |  |  | -1.58 | -1.17 | -1.32 | -1.13 | -1.74 | -0.84 |  |
| -1.55 | -0.87 | 2.00 |  |  |  |  | -1.54 | -1.10 |  |  | -1.59 | -1.18 | -1.45 | -1.16 | -1.85 | -0.88 |  |
| -1.75 | -0.98 | 2.25 |  |  |  |  | -1.56 | -1.08 |  |  | -1.59 | -1.18 | -1.58 | -1.24 | -2.01 | -1.02 |  |
| -1.94 | -1.09 | 2.50 |  |  |  |  | -1.61 | -1.14 |  |  | -1.76 | -1.22 | -1.62 | -1.30 | -2.10 | -1.09 |  |
| -2.13 | -1.19 | 2.75 |  |  |  |  |  |  |  |  | -1.84 | -1.34 |  |  | -2.28 | -1.17 |  |
| -2.33 | -1.30 | 3.00 |  |  |  |  |  |  |  |  | -2.00 | -1.39 |  |  | -2.42 | -1.17 |  |
| -2.52 | -1.41 | 3.25 |  |  |  |  |  |  |  |  | -2.14 | -1.46 |  |  | -2.59 | -1.19 |  |
| -2.72 | -1.52 | 3.50 |  |  |  |  |  |  |  |  | -2.32 | -1.42 |  |  | -2.63 | -1.23 |  |
| -2.91 | -1.63 | 3.75 |  |  |  |  |  |  |  |  | -2.41 | -1.39 |  |  | -2.69 | -1.17 |  |

Center coordinates of left main bronchus in Adults (QS)

| Model | | | Actual | | | | | | | | | | | | | | |
| --- | --- | --- | --- | --- | --- | --- | --- | --- | --- | --- | --- | --- | --- | --- | --- | --- | --- |
| Adults | | | 14 | | 15 | | 16 | | 17 | | 18 | | 19 | | 20 | |  |
| L-R | A-P | S-I | L-R | A-P | L-R | A-P | L-R | A-P | L-R | A-P | L-R | A-P | L-R | A-P | L-R | A-P |  |
| 0.00 | 0.00 | 0.00 | 0.00 | 0.00 | 0.00 | 0.00 | 0.00 | 0.00 | 0.00 | 0.00 | 0.00 | 0.00 | 0.00 | 0.00 | 0.00 | 0.00 |  |
| 0.25 | -0.09 | 0.25 | 0.35 | -0.16 | 0.49 | 0.05 | 0.54 | -0.02 | 1.02 | -0.34 | 0.65 | -0.19 | 0.85 | -0.01 | 0.53 | -0.11 |  |
| 0.51 | -0.17 | 0.50 | 0.47 | -0.19 | 0.56 | 0.00 | 0.71 | -0.06 | 1.27 | -0.57 | 0.83 | -0.23 | 1.01 | -0.18 | 0.88 | -0.27 |  |
| 0.76 | -0.26 | 0.75 | 0.63 | -0.26 | 0.80 | -0.04 | 0.80 | -0.17 | 1.53 | -0.73 | 1.00 | -0.31 | 1.33 | -0.34 | 0.98 | -0.42 |  |
| 1.01 | -0.35 | 1.00 | 0.74 | -0.26 | 1.02 | -0.09 | 1.06 | -0.28 | 1.86 | -0.92 | 1.24 | -0.40 | 1.53 | -0.42 | 1.28 | -0.52 |  |
| 1.27 | -0.43 | 1.25 | 0.95 | -0.42 | 1.22 | -0.16 | 1.47 | -0.47 | 2.29 | -1.03 | 1.48 | -0.44 | 1.86 | -0.50 | 1.39 | -0.61 |  |
| 1.52 | -0.52 | 1.50 | 1.37 | -0.61 | 1.51 | -0.33 | 1.49 | -0.58 | 2.47 | -1.13 | 1.78 | -0.60 | 2.24 | -0.64 | 1.57 | -0.70 |  |
| 1.78 | -0.60 | 1.75 | 1.72 | -0.75 | 1.80 | -0.48 | 1.60 | -0.60 |  |  | 1.84 | -0.68 | 2.45 | -0.71 | 1.70 | -0.77 |  |
| 2.03 | -0.69 | 2.00 | 2.14 | -0.93 | 2.04 | -0.55 | 1.64 | -0.64 |  |  | 1.94 | -0.75 | 3.06 | -0.77 | 2.00 | -0.84 |  |
| 2.28 | -0.78 | 2.25 | 2.61 | -1.17 | 2.36 | -0.70 | 1.66 | -0.69 |  |  | 2.07 | -0.89 | 3.32 | -0.66 | 2.64 | -0.91 |  |
| 2.54 | -0.86 | 2.50 | 2.82 | -1.26 | 2.55 | -0.77 | 1.86 | -0.75 |  |  | 2.56 | -0.93 |  |  |  |  |  |
| 2.79 | -0.95 | 2.75 | 3.20 | -1.43 | 2.94 | -0.87 | 2.29 | -0.97 |  |  | 3.27 | -0.98 |  |  |  |  |  |
| 3.04 | -1.04 | 3.00 |  |  | 3.37 | -1.03 | 3.12 | -1.34 |  |  |  |  |  |  |  |  |  |
| 3.30 | -1.12 | 3.25 |  |  |  |  | 4.20 | -1.57 |  |  |  |  |  |  |  |  |  |
